# USP53 Drives Ethanol-Induced Myocardial Injury by Promoting K63 Deubiquitination-Dependent RIPK1 Activation at K377

**DOI:** 10.34133/research.0823

**Published:** 2025-08-14

**Authors:** Jichen Pan, Xiaolin Liu, Xiao Li, Shanshan Wang, Yuliang Zhao, Chong Yuan, Dongdong Liu, Liyan Wang, Meng Zhang, Fengming Liu, Mei Zhang, Shen Dai

**Affiliations:** ^1^ State Key Laboratory for Innovation and Transformation of Luobing Theory; Key Laboratory of Cardiovascular Remodeling and Function Research of MOE, NHC, CAMS and Shandong Province; Department of Cardiology, Qilu Hospital of Shandong University, Jinan 250012, China.; ^2^ School of Basic Medical Sciences, Shandong University, Jinan 250012, China.; ^3^ Morphological Experimental Center, School of Basic Medical Sciences, Shandong University, Jinan 250012, China.

## Abstract

Alcoholic cardiomyopathy (ACM) is a type of dilated cardiomyopathy unrelated to ischemia, which develops as a consequence of chronic alcohol consumption. While ethanol-induced irreversible cardiomyocyte death is implicated in ACM development and progression, the precise molecular mechanisms involved are still obscure. In the current study, we demonstrate that ethanol exposure promotes receptor-interacting serine/threonine-protein kinase 1 (RIPK1) autophosphorylation and enhances pRIPK1-associated apoptosis and necroptosis in ACM models both in vivo and in vitro. Through co-immunoprecipitation (Co-IP) combined with liquid chromatography–tandem mass spectrometry (LC-MS/MS) analysis, we identified ubiquitin-specific protease 53 (USP53) as a pivotal deubiquitinase involved in modulating RIPK1 activation following ethanol stimulation in cardiomyocytes. Mechanistically, we found that ethanol induced up-regulation of USP53 via transcriptional induction by early growth response 1 (EGR1). Subsequently, USP53 interacted with the intermediate domain of RIPK1 and removed K63-linked ubiquitination at lysine-377 (K377), facilitating RIPK1 phosphorylation and triggering downstream apoptotic and necroptotic pathways in cardiac cells. Further, alcohol-fed cardiomyocyte-specific USP53 knockout (USP53^CKO^) mice exhibited improved survival rates and less cardiomyocyte death in hearts compared with the control group. Our study identifies USP53 as a novel regulator of RIPK1-dependent cell death and advances our understanding of the mechanistic pathways of ACM. These results highlight the USP53–RIPK1 signaling axis as a potential therapeutic target for mitigating ACM progression.

## Introduction

Alcoholic cardiomyopathy (ACM) is defined as chronic and excessive ethanol (EtOH) intake-induced dilated cardiomyopathy, which is characterized by cardiac chamber enlargement, increased interstitial fibrosis, and impaired cardiac contractility [1,2]. Given that targeted treatments are absent, abstinence is the only therapeutic option for patients with ACM. Both EtOH and its metabolite, such as acetaldehyde, can induce mitochondrial dysfunction and promote oxidative damage in cardiomyocytes (CMs). These pathological processes ultimately lead to cellular impairment and programmed cell death. Clinical studies and animal models have shown that alcohol consumption leads to an elevation of apoptosis in CMs [3–5]. In addition, recent researches revealed the up-regulation of necroptosis [6–8] and ferroptosis [9,10] in EtOH-stimulated CMs. Multiple forms of cell death can coexist and cross-regulate each other, collectively driving disease progression. The death of CMs is a major factor in ventricular remodeling and heart failure [11]. Determining the predominant type of cell death contributing to ACM development is crucial for understanding the underlying molecular pathways and identifying therapeutic targets.

Receptor-interacting protein kinase 1 (RIPK1) functions as a crucial mediator in controlling inflammatory responses and cell death pathways, including apoptosis, necroptosis, and pyroptosis, under certain conditions [12]. Upon tumor necrosis factor (TNF) or reactive oxygen species (ROS) stimulation, RIPK1 is promptly directed to form transient membrane-bound complex I, where RIPK1 is heavily ubiquitinated by cIAP1/2 to trigger the nuclear factor κB (NF-κB) pathway and cell survival [13,14]. Dysregulation of the posttranslational modification of RIPK1, such as dissociation of certain ubiquitin, will induce its kinase activation and subsequently autophosphorylation, which is characterized by phosphorylation at serine-166. These molecular events promote RIPK1 to participate in the assembly of cytosolic complex II, where it serves as a scaffold for caspase-8 binding and activation, ultimately initiating the apoptotic cascade [15]. On the other hand, cell death signaling could also skew toward necroptosis, in which RIPK1 binds receptor-interacting protein kinase 3 (RIPK3) through RHIM domain (RIP homotypic interaction motif), subsequently leading to mixed lineage kinase domain like (MLKL) activation [16]. Given the pivotal role in determining cell fate, RIPK1 is tightly regulated to prevent adverse effects in cells. Various posttranslational modifications act as death checkpoints to suppress the cytotoxic effect of RIPK1, such as ubiquitination, deubiquitination, and phosphorylation [17,18]. In recent years, studies revealed that the dysregulation of ubiquitination and deubiquitination of RIPK1 is closely associated with the progression of inflammatory disorders, neurodegeneration, and oncological diseases. However, the involvement and underlying mechanism of RIPK1 activation in alcoholic cardiac toxicity have not been reported.

In this study, we identified ubiquitin-specific protease 53 (USP53) as a crucial modulator that induces the RIPK1 kinase activation and subsequent programmed cell death in ACM progression. As a member of the USP family, USP53 is broadly expressed in cardiac tissue. Notably, it has been considered catalytically inactive due to the lack of an essential histidine [19,20]. However, recent studies found that USP53 is a robust deubiquitinating enzyme (DUB) and preferentially catalyzes K63-linked ubiquitin chain. In addition, the study indicates that USP53 promotes the development of atherosclerosis by stabilizing the scavenger receptor-A protein via the removal of the K48-linked ubiquitin chain. [21]. Furthermore, the overexpression of USP53 has been reported to induce cell death in various cancers [22–25], which indicates that USP53 is closely related to cell death regulation. Here, we found that alcohol led to the up-regulation of USP53 in CMs. USP53 directly binds to RIPK1 and triggers its kinase activity by removing the K63-linked ubiquitin chain at the lysine-377 (K377) site, which in turn drives apoptosis and necroptosis, and exacerbates ACM. These findings revealed the critical role of USP53–RIPK1 interaction in driving alcoholic CM injury and ACM. In the meantime, it highlights USP53 as a promising therapeutic target in ACM treatment.

## Results

### EtOH contributes to cardiac dysfunction and triggers both apoptosis and necroptosis in CMs

**Fig. 1.**
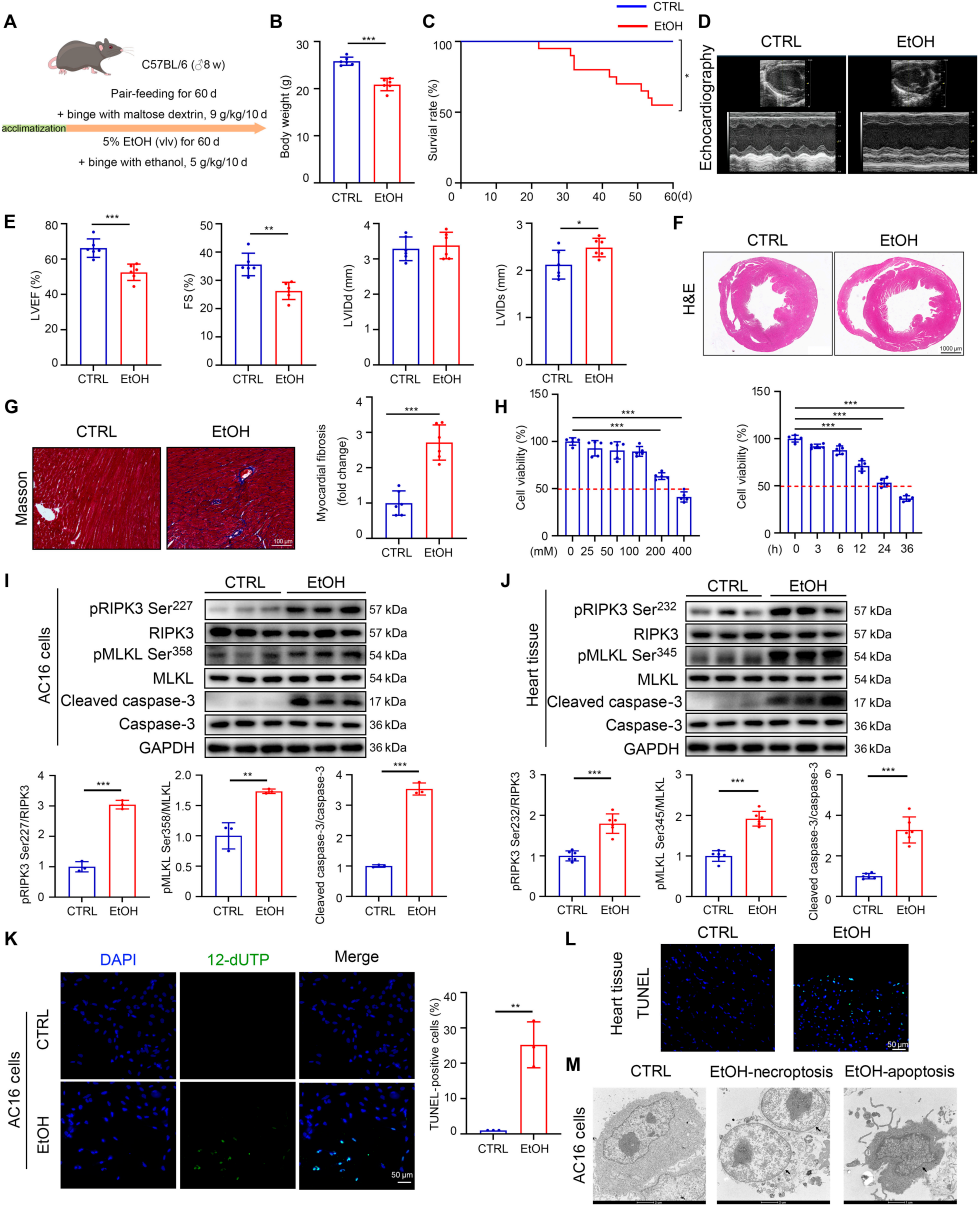
EtOH contributes to cardiac dysfunction and triggers both apoptosis and necroptosis in CMs. (A) Experimental procedure of the ACM mice model. (B) Body weights of the mice in the CTRL and EtOH groups (*n* = 6 each). (C) Survival rate of the mice in the CTRL and EtOH groups. (D and E) Transthoracic echocardiographic results of the LVEF, FS, LVIDd, and LVIDs for cardiac functional analysis (*n* = 6 each). (F) Representative images of heart tissues stained with H&E (scale bars, 1,000 μm). (G) Representative images of heart tissues stained with Masson (*n* = 6 each) (scale bars, 100 μm). (H) Cell viability of AC16 cells following EtOH treatment was evaluated at varying concentrations and differential time points by CCK8 assay (*n* = 5 each). (I) Western blotting of pRIPK3 Ser^227^, RIPK3, pMLKL Ser^358^, MLKL, cleaved caspase-3, and caspase-3 in EtOH-treated AC16 cells and CTRL (*n* = 3 each). (J) Western blotting of pRIPK3 Ser^232^, RIPK3, pMLKL Ser^345^, MLKL, cleaved caspase-3, and caspase-3 in heart tissues from EtOH-fed mice and CTRL (*n* = 6 each). (K) TUNEL staining of EtOH-treated AC16 cells and CTRL (*n* = 3 each) (scale bars, 50 μm). (L) TUNEL staining of heart tissue from EtOH-fed mice and CTRL (scale bars, 50 μm). (M) TEM assay of AC16 cells. Necroptotic cells exhibit nuclear swelling, whereas apoptotic cells are characterized by nuclear shrinkage (arrowheads). Results are expressed as the mean ± SD. *P < 0.05, **P < 0.01, ***P < 0.001.

After 60 d of EtOH feeding (Fig. 1A), mice exhibited lower body weights and worse survival compared to the control (CTRL) group (Fig. 1B and C). Meanwhile, alcohol-fed mice displayed obvious symptoms of ACM characterized by marked cardiac dysfunction, including dramatically reduced left ventricular ejection fraction (LVEF) and fractional shortening (FS), accompanied by the increase of left ventricular internal diameter at the end of systole (LVIDs). The left ventricular internal diameter at the end of diastole (LVIDd) was unchanged (Fig. 1D and E)*.* Hematoxylin and eosin (H&E) staining of hearts from EtOH-fed mice showed much thinner ventricular walls and dilated cardiac chambers (Fig. 1F and Fig. S1A). Further staining with Masson revealed pronounced myocardial fibrosis and increased collagen content in the heart of the EtOH-fed group (Fig. 1G). In addition, immunohistochemistry (IHC) staining suggested that interleukin-6 (IL-6) and IL-1β were markedly elevated in the hearts from EtOH-exposed mice (Fig. S1B). ROS alterations were analyzed through dihydroethidium (DHE) staining, and the result indicated that EtOH feeding increased DHE staining obviously (Fig. S1C). These findings indicate that EtOH causes cardiac damage and ACM development.

CM injury and programmed cell death are the major causes of cardiac dysfunction and remodeling. Then, we assessed CM survival rate under EtOH exposure in vitro. Cell Counting Kit-8 (CCK8) assay observed a concentration- and time-dependent reduction in the viability of EtOH-challenged AC16 cells (Fig. 1H). To identify the predominant form of cell death involved in alcohol-related cardiac toxicity, we assessed markers for cell death pathways. Western blotting indicated that EtOH stimulation led to up-regulation of necroptosis (pRIPK3 Ser^227^/RIPK3, pMLKL Ser^358^/MLKL) and apoptosis (cleaved caspase-3/caspase-3) markers in AC16 cells (Fig. 1I), while ferroptosis and pyroptosis markers were unchanged (Fig. S1D and E). Consistent with the in vitro findings, heart tissues from EtOH-fed mice also exhibited elevated expression of necroptosis and apoptosis markers, as demonstrated by both Western blotting and immunohistochemical staining (Fig. 1J and Fig. S1F). In addition, terminal deoxynucleotidyl transferase–mediated deoxyuridine triphosphate nick end labeling (TUNEL) assay revealed significantly elevated apoptosis in EtOH-challenged AC16 cells and heart tissue coming from alcohol-fed mice (Fig. 1K and L). Transmission electron microscopy (TEM) assay analysis of AC16 cells 24 h after EtOH stimulation clearly showed increased apoptosis and necroptosis of cells (Fig. 1M). The typical visual pattern of necroptosis identified in the TEM images included swollen nuclei and mitochondria, plasma membrane disruption, and leakage of cellular components, while apoptosis was characterized as nuclear shrinkage, chromatin condensation, and apoptotic body formation.

### Autophosphorylation of RIPK1 mediates alcoholic apoptosis and necroptosis of CMs in vivo and in vitro

**Fig. 2.**
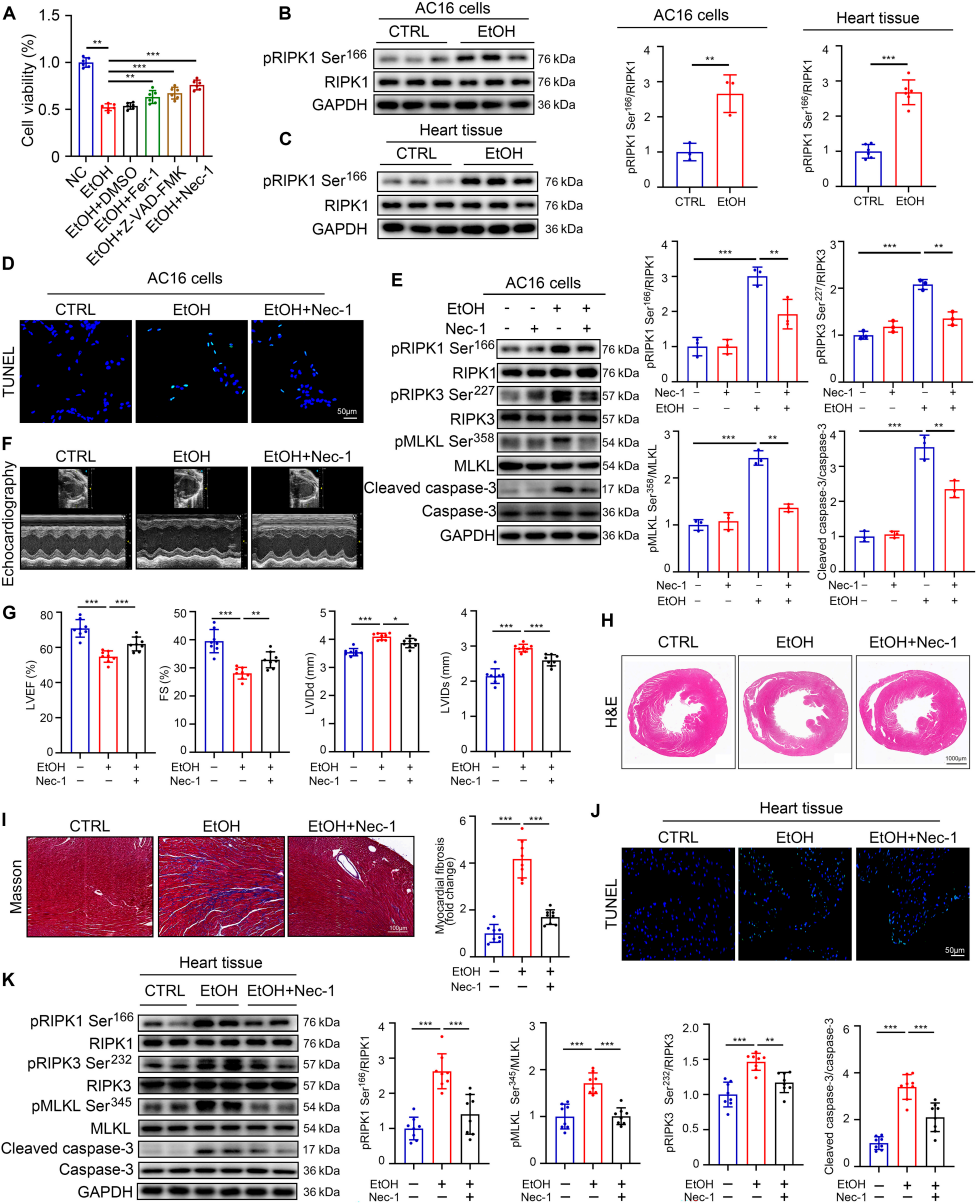
Autophosphorylation of RIPK1 mediates alcoholic apoptosis and necroptosis of CMs in vivo and in vitro. (A) Cell viability of AC16 cells treated with EtOH and subsequently rescued using Fer-1, Z-VAD-FMK, or Nec-1 was determined using the CCK8 assay (*n* = 6 each). (B) Western blotting of pRIPK1 Ser^166^ and RIPK1 in AC16 cells treated with EtOH (*n* = 3 each). (C) Western blotting of pRIPK1 Ser^166^ and RIPK1 in heart tissues from EtOH-fed mice and CTRL (*n* = 6 each). (D) TUNEL staining of AC16 cells from CTRL, EtOH, and EtOH + Nec-1 groups (*n* = 3 each) (scale bars, 50 μm). (E) Western blotting of pRIPK1 Ser^166^, RIPK1, pRIPK3 Ser^227^, RIPK3, pMLKL Ser^358^, MLKL, cleaved caspase-3, and caspase-3 in AC16 cells of the CTRL, EtOH, and EtOH + Nec-1 groups (*n* = 3 each). (F and G) Transthoracic echocardiographic results of the LVEF, FS, LVIDd, and LVIDs for cardiac functional analysis (*n* = 8 each). (H) Representative images of heart tissues stained with H&E staining (scale bars, 1,000 μm). (I) Representative images of heart tissues stained with Masson (*n* = 8 each) (scale bars, 100 μm). (J) TUNEL staining of heart tissues of the mice from CTRL, EtOH, and EtOH + Nec-1 groups (scale bars, 50 μm). (K) Western blotting of pRIPK1 Ser^166^, RIPK1, pRIPK3 Ser^232^, RIPK3, pMLKL Ser^345^, MLKL, cleaved caspase-3, and caspase-3 in heart tissues of the mice in the CTRL, EtOH, and EtOH + Nec-1 groups (*n* = 8 each). Results are expressed as the mean ± SD. *P < 0.05, **P < 0.01, ***P < 0.001.

To identify the major death type involved in EtOH-mediated CM injury, we investigated the effects of multiple cell death inhibitors on EtOH-treated AC16 cells, including Z-VAD-FMK (the pan-caspase apoptosis inhibitor), necrostatin-1 (Nec-1; the necroptosis inhibitor), and ferrostatin-1 (Fer-1; the ferroptosis inhibitor). According to the CCK8 assay, Nec-1 treatment exhibited the best rescue effect (Fig. 2A). Considering that Nec-1 targets RIPK1, which is a critical upstream regulator of apoptotic and necroptotic signaling, we further studied RIPK1 expression and its autophosphorylation. We found the activation of RIPK1 (pRIPK1 Ser^166^) was dramatically up-regulated in EtOH-stimulated AC16 cells and heart tissue from alcohol-fed mice, but the protein level of total RIPK1 was unchanged (Fig. 2B and C). Further, TUNEL staining and TEM assay suggested that Nec-1 reduced apoptosis, as well as necroptosis, of AC16 cells under EtOH stimulation (Fig. 2D and Fig. S2A and B). In the meantime, we found that Nec-1 effectively inhibited EtOH-induced RIPK1 autophosphorylation, as well as the activation of key markers (pRIPK3 Ser^227^/RIPK3, pMLKL Ser^358^/MLKL, cleaved caspase-3/caspase-3) of both apoptosis and necroptosis pathways in AC16 cells (Fig. 2E). Notably, administration of Nec-1 in ACM mouse model markedly improved the survival rate of EtOH-fed mice, but did not affect mouse body weight (Fig. S2C and D). Echocardiography revealed that Nec-1 treatment reversed EtOH-induced decreases of LVEF and FS, as well as increases of LVIDd and LVIDs in vivo (Fig. 2F and G). Histopathological analysis using H&E and Masson staining suggested that Nec-1 also effectively inhibited EtOH-induced cardiac remodeling (Fig. 2H and Fig. S2E) and fibrosis (Fig. 2I). TUNEL staining suggested that Nec-1 treatment inhibited apoptosis in heart tissue of EtOH-fed mice (Fig. 2J). RIPK1 phosphorylation, as well as the activation of necroptosis and apoptosis pathways, was also inhibited in the heart of alcohol-fed mice (Fig. 2K). These data indicated EtOH-induced apoptosis and necroptosis of CMs by promoting RIPK1 phosphorylation, which can be reversed by Nec-1 both in vitro and in vivo.

### USP53 is the critical regulator for alcohol-induced RIPK1 phosphorylation and CM death

**Fig. 3.**
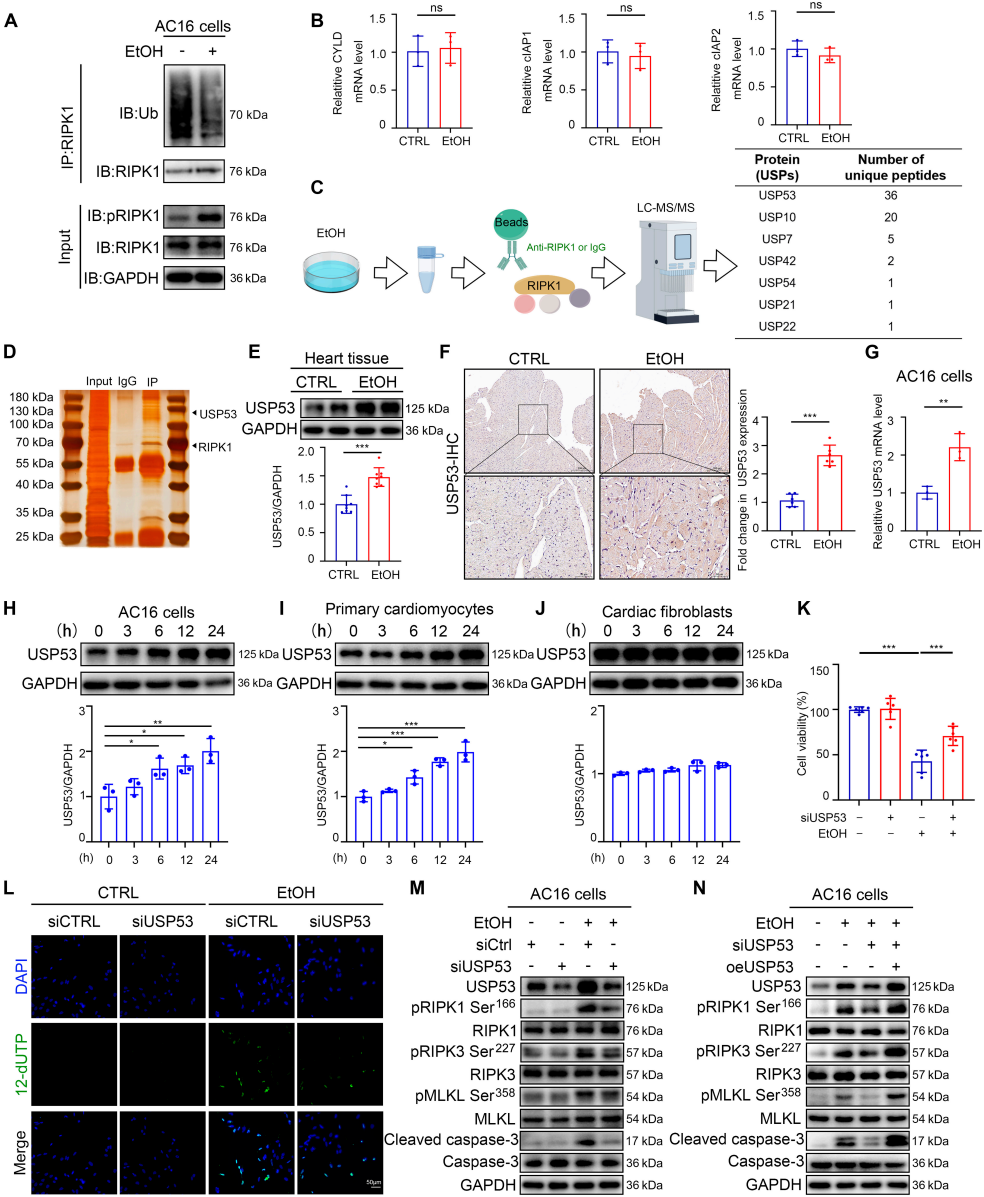
USP53 is the critical regulator for alcohol-induced RIPK1 phosphorylation and CM death. (A) Ubiquitination level of immunoprecipitated RIPK1 in EtOH-treated AC16 cells and CTRL. (B) PCR analysis of CYLD, cIAP1, and cIAP2 transcription in EtOH-treated AC16 cells (*n* = 3 each). (C) Schematic view and results of Co-IP coupled with LC-MS/MS. (D) Silver staining of proteins associated with immunoprecipitated RIPK1. (E) Western blotting for USP53 in heart tissues from EtOH-fed mice and CTRL mice (*n* = 8 each). (F) Representative images of IHC staining of USP53 in heart tissues from EtOH-fed mice and CTRL mice. (G) PCR analysis of USP53 transcription in EtOH-treated AC16 cells (*n* = 3 each). (H) Western blotting of USP53 in the AC16 cells following EtOH treatment at different time points (*n* = 3 each). (I) Western blotting of USP53 in the primary CMs following EtOH treatment at different time points (*n* = 3 each). (J) Western blotting of USP53 in the CFs following EtOH treatment at different time points (*n* = 3 each). (K) CCK8 assays of AC16 cells transfected with USP53-siRNA and treated with EtOH (*n* = 6 each). (L) TUNEL staining of AC16 cells transfected with USP53-siRNA and treated with EtOH (*n* = 3 each). (M and N) Western blotting of pRIPK1 Ser^166^, RIPK1, pRIPK3 Ser^227^, RIPK3, pMLKL Ser^358^, MLKL, cleaved caspase-3, and caspase-3 in the AC16 cells transfected with USP53-siRNA and treated with EtOH (M; *n* = 3 each), or USP53-deficient AC16 cells that were treated with EtOH, with or without USP53 reconstitution (N; *n* = 3 each). Results are expressed as the mean ± SD. *P < 0.05, **P < 0.01, ***P < 0.001.

Given that the ubiquitylation level is critical in determining the involvement of RIPK1 in NF-κB signaling, apoptotic, or necroptotic pathways, we thought to clarify whether EtOH-induced pRIPK1 up-regulation in CMs is intricately associated to ubiquitination modification through IP assay first. The result indicated that alcohol markedly inhibited the ubiquitination of RIPK1 in AC16 cells (Fig. 3A). Then, we assessed the widely studied deubiquitinating enzyme and E3 ligases of RIPK1, and found that EtOH did not affect the expression of CYLD, cIAP1, and cIAP2 at the RNA level (Fig. 3B). Next, we use Co-IP and liquid chromatography–mass spectrometry/mass spectrometry (LC-MS/MS) assay to search for the regulator of RIPK1 ubiquitination under EtOH stimulation. By analyzing the profile of precipitated proteins, USP53 was ultimately selected for further evaluation since the high binding affinity of RIPK1 (Fig. 3C). The silver staining revealed the bands of RIPK1-bound proteins (Fig. 3D). Western blotting and IHC staining suggested that USP53 expression was markedly increased in the heart of EtOH-exposed mice (Fig. 3E and F). Meanwhile, EtOH significantly promoted the expression of USP53 in AC16 cells at RNA level (Fig. 3G). Notably, the up-regulation of USP53 in EtOH-exposed AC16 cells and rat primary CMs occurred in the time- and concentration-dependent manner, but was not observed in cardiac fibroblasts (CFs) (Fig. 3H to J and Fig. S3A to C).

Next, we evaluated whether USP53 was incorporated in RIPK1 phosphorylation and pRIPK1-associated cell death pathways in EtOH-treated CMs. By knockdown of USP53 by small interfering RNA (siRNA) (Fig. S3D), we found that USP53 deficiency markedly rescued EtOH-induced decrease in AC16 cell survival (Fig. 3K). Meanwhile, TUNEL staining suggested that USP53-deficient AC16 cells exhibited a lower level of apoptosis under EtOH challenge (Fig. 3L and Fig. S3E). Notably, Western blotting showed that USP53 knockdown dramatically reversed the up-regulation of pRIPK1 Ser^166^, pRIPK3 Ser^227^, pMLKL Ser^358^, and cleaved caspase-3 levels in EtOH-challenged AC16 cells (Fig. 3M and Fig. S3F). In contrast, overexpression of USP53 significantly decreased the survival and proliferation of AC16 cells under EtOH treatment (Fig. S3G and H). In addition, USP53 overexpression significantly reversed USP53 loss-mediated suppression effect on apoptosis and necroptosis pathways (Fig. 3N and Fig. S3I). Thus, these data indicated that USP53 participated in EtOH-induced myocardial injury by modulating RIPK1 autophosphorylation.

### USP53 interacts with the intermediate domain of RIPK1 via its C-terminal region

**Fig. 4.**
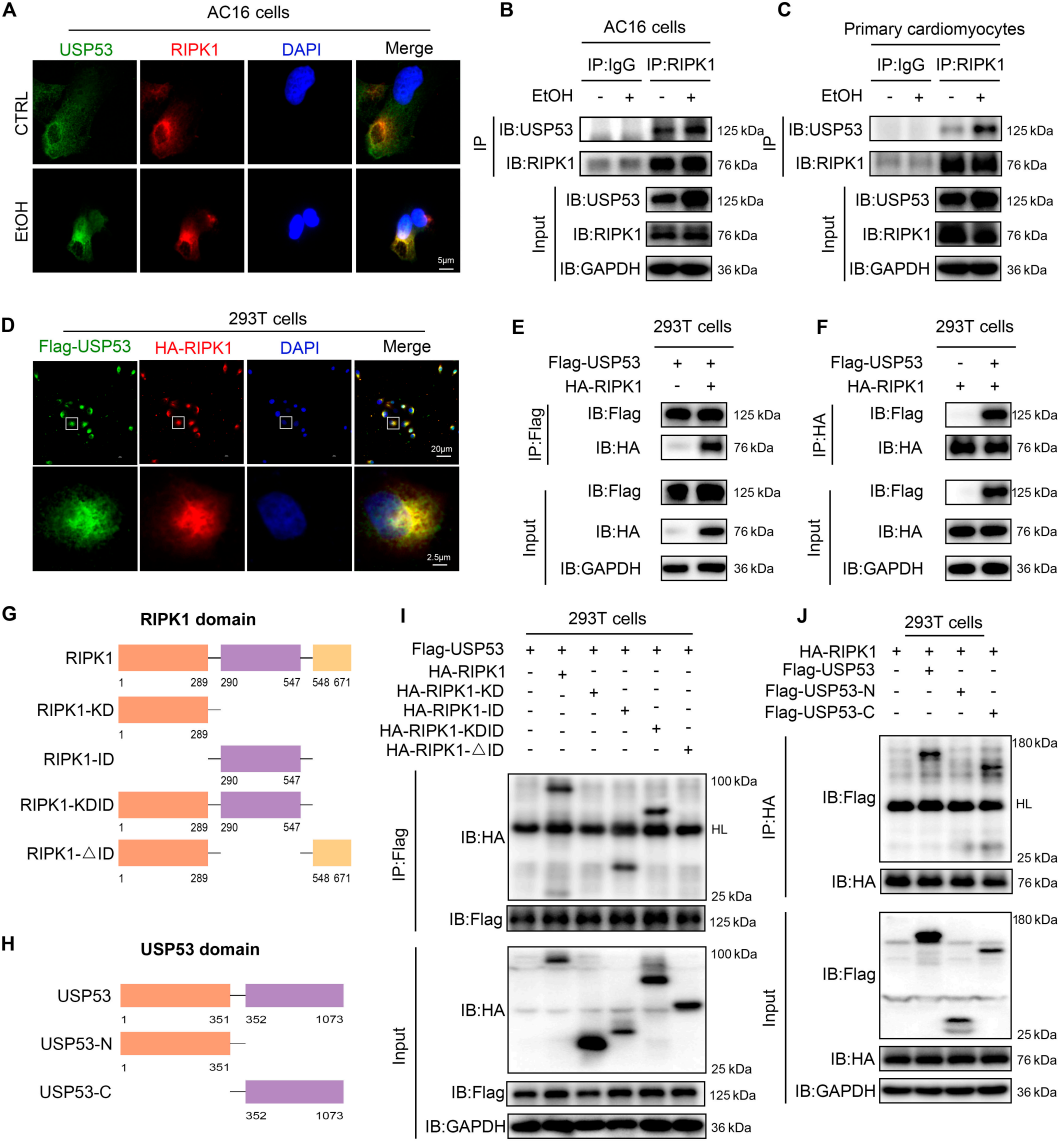
USP53 can directly bind to the intermediate domain of RIPK1 through its C terminus. (A) Representative fluorescence microscopy images of RIPK1 (red) and USP53 (green) in EtOH-treated AC16 cells and CTRL cells (scale bars, 5 μm). (B and C) Co-IP analysis of the endogenous interaction of USP53 with RIPK1 in EtOH-treated AC16 cells and primary CMs. (D) Representative fluorescence microscopy images for HA (red) and Flag (green) in HEK-293T cells transfected with Flag-USP53 and HA-RIPK1 (scale bars, 20 and 5 μm). (E and F) Co-IP analysis of exogenous interaction of USP53 and RIPK1 in HEK-293T cells transfected with Flag-USP53 and HA-RIPK1. (G) Schematic diagram of RIPK1 and its truncation mutants. (H) Schematic diagram of USP53 and its truncation mutants. (I) Cotransfected Flag-USP53 and HA-RIPK1 or its mutants into HEK-293T cells. Cell lysates were subjected to anti-Flag IP before Western blotting. (J) Cotransfected HA-RIPK1 and Flag-USP53 or its mutants into HEK-293T cells. Cell lysates were subjected to anti-HA IP before Western blotting.

Immunofluorescence assay indicated colocalization of RIPK1 and USP53 in the cytoplasm of AC16 cells with EtOH treatment (Fig. 4A). Co-IP assay suggested direct binding between RIPK1 and USP53 in AC16 cells and primary rat CMs, and the binding was dramatically enhanced under EtOH treatment, accompanied with increased USP53 expression (Fig. 4B and C). Similar results were found in human embryonic kidney (HEK)-293T cells transfected with hemagglutinin (HA)-RIPK1 and Flag-USP53, which showed colocalization of HA-RIPK1 and Flag-USP53 in cytoplasm (Fig. 4D). Then, we immunoprecipitated either Flag or HA tag and probed for both proteins. The results showed direct interaction between HA-RIPK1 and Flag-USP53 (Fig. 4E and F). Given that RIPK3 is also a central mediator of necroptosis and is known to undergo ubiquitin modification, we performed Co-IP assays to investigate whether USP53 directly interacts with RIPK3. The results did not show detectable interaction between USP53 and RIPK3 in AC16 cells, either with or without EtOH treatment. These findings suggest that USP53 primarily functions through the interaction with RIPK1, rather than RIPK3 (Fig. S4A).

To identify the essential domain that RIPK1 required to bind with USP53, we constructed 4 HA-tagged truncation mutants of RIPK1 based on its well-known structure, including HA-RIPK1-KD (kinase domain), HA-RIPK1-ID (intermediate domain), HA-RIPK1-KDID (kinase domain and intermediate domain), and HA-RIPK1-ΔID (intermediate domain-depleted RIPK1) (Fig. 4G). By cotransfection of Flag-tagged USP53 and each RIPK1 mutant in 293T cells, we found that HA-RIPK1, KDID domain, and ID domain showed obvious interaction with USP53, but not KD domain or RIPK1 lacking ID domain (Fig. 4I). These findings indicate the ID domain of RIPK1 as a crucial structural element for USP53 interaction. Furthermore, to identify the critical binding region of USP53 required for its association with RIPK1, we constructed 2 Flag-tagged truncation mutants of USP53, including its C-terminal domain (Flag-USP53-C) and N-terminal domain (Flag-USP53-N) (Fig. 4H). Cotransfection of USP53 mutants and HA-tagged RIPK1 in HEK-293T cells showed that both the full length and the C terminus of USP53 could interact with RIPK1, but not the N terminus of USP53, which indicated that the C terminus of USP53 was essential for interacting with RIPK1 (Fig. 4J).

### USP53 deubiquitinates K63-linked ubiquitin chain of RIPK1 at K377 promoting RIPK1 phosphorylation and CM injury

**Fig. 5.**
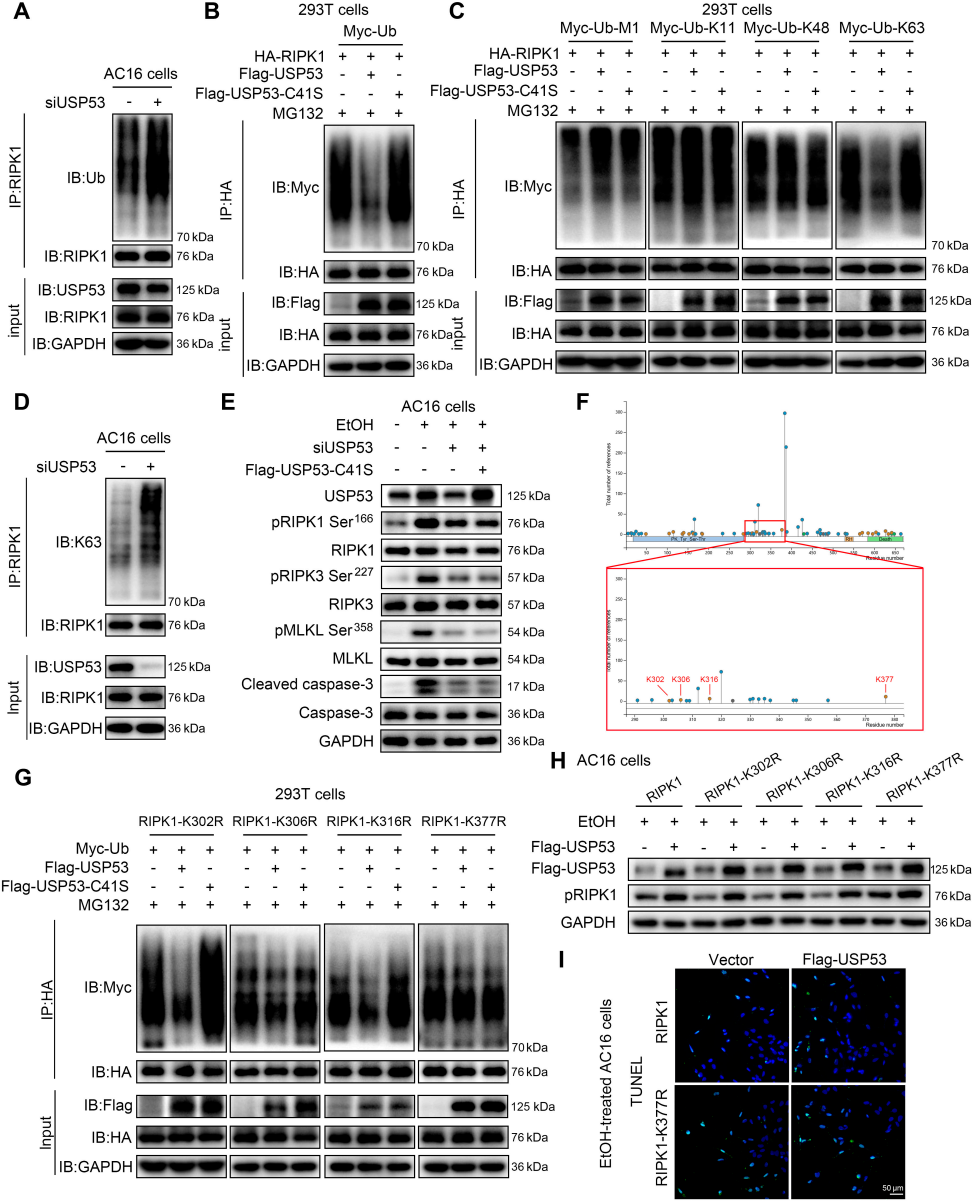
USP53 deubiquitinates K63-linked ubiquitin chain of RIPK1 at K377 promoting RIPK1 phosphorylation and CM injury. (A) Ubiquitination of immunoprecipitated RIPK1 in AC16 cells transfected with USP53-siRNA or CTRL. (B) Exogenous ubiquitination assays of HA-RIPK1 in HEK-293T cells cotransfected with Myc-Ub, HA-RIPK1, and Flag-USP53 or Flag-USP53-C41S. (C) Exogenous ubiquitination assays of HA-RIPK1 in HEK-293T cells cotransfected with HA-RIPK1 and ubiquitin variants, including Myc-Ub-M1, Myc-Ub-K11, Myc-Ub-K48, or Myc-Ub-K63 in the presence of Flag-USP53 or Flag-USP53-C41S. (D) K63 ubiquitination of immunoprecipitated RIPK1 was analyzed by Western blotting in AC16 cells transfected with USP53-siRNA. (E) Western blotting of markers in necroptosis and apoptosis pathways of USP53-deficient AC16 cells under EtOH treatment, with or without USP53-C41S reconstitution (*n* = 3 each). (F) Schematic diagram of ubiquitination sites on the ID of RIPK1 identified from PhosphosSitePlus (www.phosphosite.org). (G) Exogenous ubiquitination assays of lysine residue mutants of RIPK1 in HEK-293T cells. HEK-293T cells were cotransfected with Myc-Ub and lysine residue mutants of RIPK1 in the presence of Flag-USP53 or Flag-USP53-C41S. (H) Western blotting analysis of pRIPK1 Ser^166^ in AC16 cells transfected with RIPK1 lysine residue mutants (*n* = 3 each). (I) TUNEL staining of EtOH-stimulated AC16 cells cotransfected with Flag-USP53 and RIPK1 or RIPK1-K377R mutant (*n* = 3 each) (scale bars, 50 μm).

Given that USP53 only affected RIPK1 phosphorylation (Fig. 3M), but not at the RNA and protein level (Fig. S5A to D), we thought that USP53 might regulate RIPK1 activity through posttranslational modifications, such as ubiquitination, a crucial regulator to activate RIPK1 kinase activity [26,27]. To further evaluate whether USP53 regulates the ubiquitination of RIPK1, we knocked down USP53 expression in AC16 cells by siRNA. The result indicated that the level of ubiquitination of RIPK1 was up-regulated in USP53-deficient cells (Fig. 5A). Next, to assess whether the hydrolysis of ubiquitin chains on RIPK1 requires the catalytic activity of USP53, we generated a catalytically inactive mutant, Flag-USP53-C41S, in which the critical cysteine at position 41 was substituted with serine, thereby abolishing its deubiquitinating function. By cotransfection of HA-RIPK1 with either Flag-USP53 or Flag-USP53-C41S in HEK-293T cells, we found that catalytic site-mutated USP53-C41S was unable to inhibit the ubiquitination of RIPK1 (Fig. 5B). Further, given the established involvement of M1-, K11-, K48-, and K63-linked ubiquitin chains in modulating RIPK1 activity [28], to figure out which Ub chain was preferentially catalyzed by USP53, we generated vectors of these 4 types of ubiquitin. The data demonstrated that USP53 markedly attenuated K63-linked ubiquitination of RIPK1, while no effect was observed on M1, K11, and K48 ubiquitin. In the meantime, the USP53-C41S mutant failed to suppress the K63 ubiquitination of RIPK1 (Fig. 5C). Consistently, we found that USP53 deficiency led to an increase of K63-linked ubiquitination of RIPK1 in AC16 cells, but not K48-linked ubiquitination (Fig. 5D and Fig. S5E). In the meantime, overexpression of USP53-C41S failed to reverse USP53 knockdown-induced suppression effects of apoptosis and necroptosis pathways in EtOH-exposed AC16 cells (Fig. 5E and Fig. S5F). These results suggest that USP53 displays a deubiquitination effect preferentially on K63-linked ubiquitination of RIPK1.

To further determine the modification site of K63-linked Ub on RIPK1, we identified 4 predicted ubiquitination sites (Lys^302^, Lys^306^, Lys^316^, and Lys^377^) within the ID domain of RIPK1 by the PhosphosSitePlus website (www.phosphosite.org) (Fig. 5F) and by analyzing previous studies [17,29]. We mutated lysine (K) to arginine (R) to generate point mutants of RIPK1, including HA-RIPK1-K302R, HA-RIPK1-K306R, HA-RIPK1-K316R, and HA-RIPK1-K377R. By cotransfection of Flag-USP53, Myc-Ub, and each RIPK1 mutant in HEK-293T cells, we observed that the K377R mutation effectively inhibited the USP53-induced RIPK1 deubiquitination, whereas K302R, K306R, and K316R did not affect this process (Fig. 5G). However, Co-IP assay revealed that the K377R mutation did not affect the interaction between RIPK1 and USP53 (Fig. S5G). Then, we assessed whether the K377R mutation can suppress USP53-mediated RIPK1 autophosphorylation in EtOH-treated AC16 cells. By cotransfection of Flag-USP53 and RIPK1 mutants in AC16 cells, we observed that overexpression of USP53 significantly augmented EtOH-induced RIPK1 autophosphorylation, while K377R mutation effectively blocked the up-regulation of pRIPK1 Ser^166^ mediated by USP53 (Fig. 5H and Fig. S5H). Meanwhile, the K377R mutation markedly inhibited USP53 overexpression-enhanced apoptosis in AC16 cells (Fig. 5I and Fig. S5I). These results suggest that USP53 activates RIPK1 by the deubiquitination of K63-linked Ub at K377 of RIPK1.

### EGR1 transcriptionally modulated USP53 expression in CMs under EtOH treatment

**Fig. 6.**
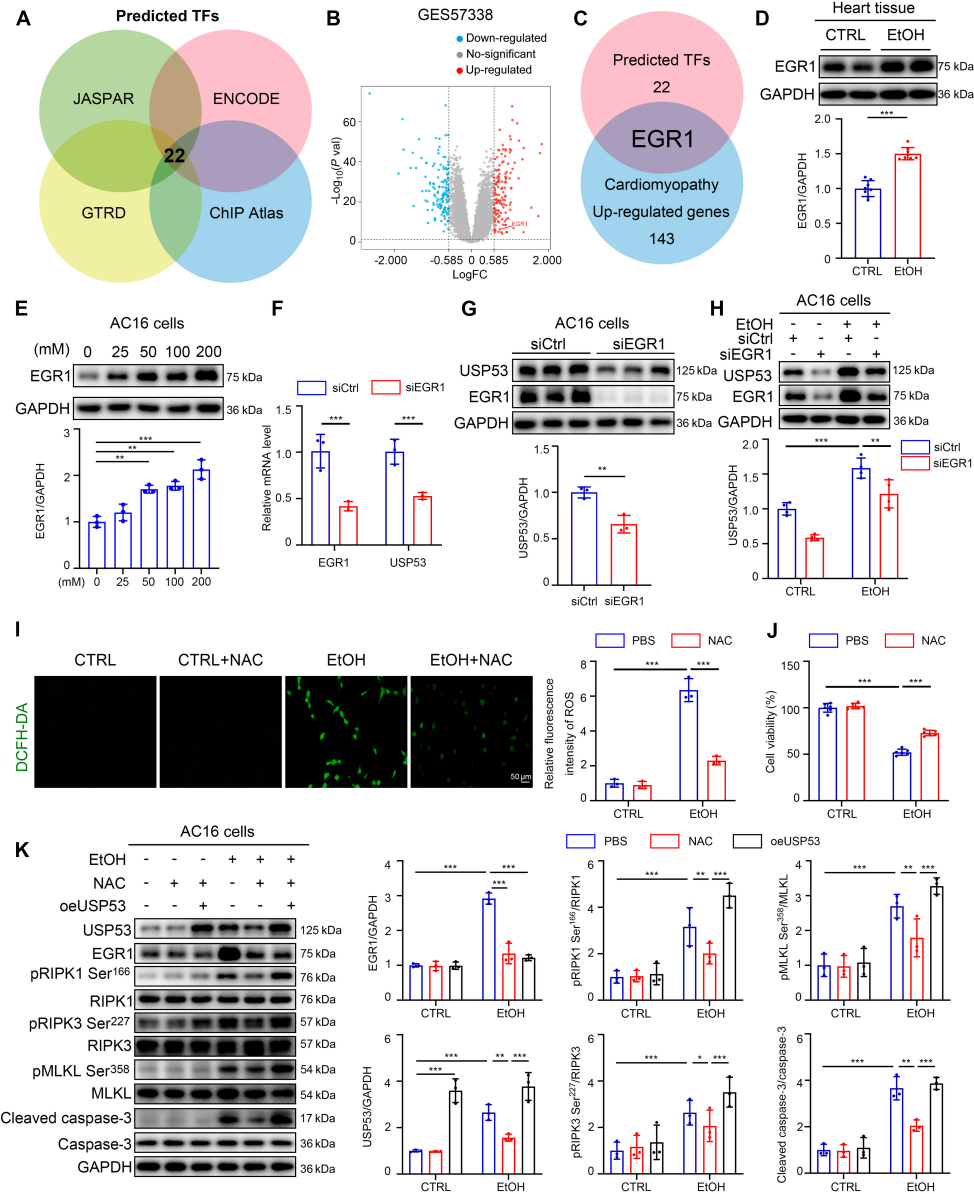
EGR1 transcriptionally modulated USP53 expression in CMs under EtOH treatment. (A) Intersection of predicted TFs of USP53 via JASPAR, ENCODE, GTRD, and ChIP Atlas. (B) Volcano plot of gene expression in GES57338 dataset. (C) Overlapping up-regulated genes in GES57338 dataset and predicted TFs. (D) Representative Western blotting for EGR1 in heart tissue of ACM mice and CTRL (*n* = 8 each). (E) Western blotting of EGR1 in the AC16 cells after EtOH treatment at a set concentration gradient (*n* = 3 each). (F) PCR analysis of EGR1 and USP53 expression in AC16 cells after transfection with EGR1-siRNA (*n* = 3 each). (G) Western blotting of EGR1 and USP53 in the AC16 cells transfected with EGR1-siRNA (*n* = 3 each). (H) Western blotting of EGR1 and USP53 in the EtOH-treated AC16 cells transfected with EGR1-siRNA (*n* = 3 each). (I) Images and analysis of AC16 cells stained with and DCFH-DA (*n* = 3 each) (scale bars, 50 μm). (J) CCK8 assay for viability of EtOH-treated AC16 cells with NAC treatment (*n* = 6 each). (K) Western blotting of EGR1, USP53, pRIPK1 Ser^166^, RIPK1, pRIPK3 Ser^227^, RIPK3, pMLKL Ser^358^, MLKL, cleaved caspase-3, and caspase-3 in the AC16 cells with NAC treatment or USP53 overexpression (*n* = 3 each). Results are expressed as the mean ± SD. *P < 0.05, **P < 0.01, ***P < 0.001.

To investigate the upstream molecular mechanisms by which EtOH elevates USP53 transcription, we employed 4 transcription factor (TF) prediction platforms—JASPAR, ChIP Atlas, GTRD, and ENCODE—to identify candidate TFs potentially binding to the USP53 promoter. Cross-analysis revealed 22 TFs commonly predicted by all 4 databases (Fig. 6A). Re-analysis of the 22 TFs and differential expressed genes (DEGs; 143 up-regulation and 136 down-regulation) from the cardiomyopathy-related Gene Expression Omnibus (GEO) dataset GSE57338 revealed early growth response 1 (EGR1) as the only overlapping molecule (Fig. 6B and C). The level of EGR1 in the heart of EtOH-fed mice was obviously up-regulated compared to that with the CTRL mice (Fig. 6D). Similarly, Western blotting suggested that EtOH treatment up-regulated EGR1 expression in AC16 cells through a dose-dependent manner (Fig. 6E). To further validate the influence of EGR1 on USP53 expression, we found that EGR1 knockdown significantly suppressed USP53 expression in AC16 cells with or without EtOH treatment (Fig. 6F to H). Notably, USP53 overexpression effectively reversed the inhibitory effects of EGR1 knockdown on pRIPK1-mediated cell death pathways (Fig. S6A). These data indicated that EGR1 is the key regulator promoting USP53 expression in CMs under EtOH treatment.

Given that ROS is reported to affect EGR1 expression [30], we next explored whether ROS was involved in EtOH-induced EGR1 up-regulation and USP53/RIPK1-mediated CM injury. 2,7-Dichlorodihydrofluorescein diacetate (DCFH-DA) staining demonstrated that EtOH treatment elevated ROS level in AC16 cells, which can be inhibited by N-acetylcysteine (NAC), an inhibitor of ROS (Fig. 6I). Furthermore, Western blotting indicated that NAC dramatically suppressed the EGR1 expression in EtOH-exposed AC16 cells (Fig. 6K). Meanwhile, NAC markedly attenuated USP53 expression and RIPK1 autophosphorylation, as well as pRIPK1-dependent apoptosis and necroptosis (Fig. 6K). Notably, USP53 overexpression effectively reversed the inhibitory effects of NAC on pRIPK1-mediated cell death pathways (Fig. 6K). Consistently, CCK8 assay showed that NAC pretreatment significantly improved the survival rate of EtOH-challenged AC16 cells (Fig. 6J). These data indicated that ROS contributes to the EtOH-induced up-regulation of EGR1 in CMs, which subsequently provokes USP53 expression and downstream RIPK1 phosphorylation and pRIPK1-dependent cell death.

### CM-specific USP53 knockout mice develop less alcoholic myocardial injury

**Fig. 7.**
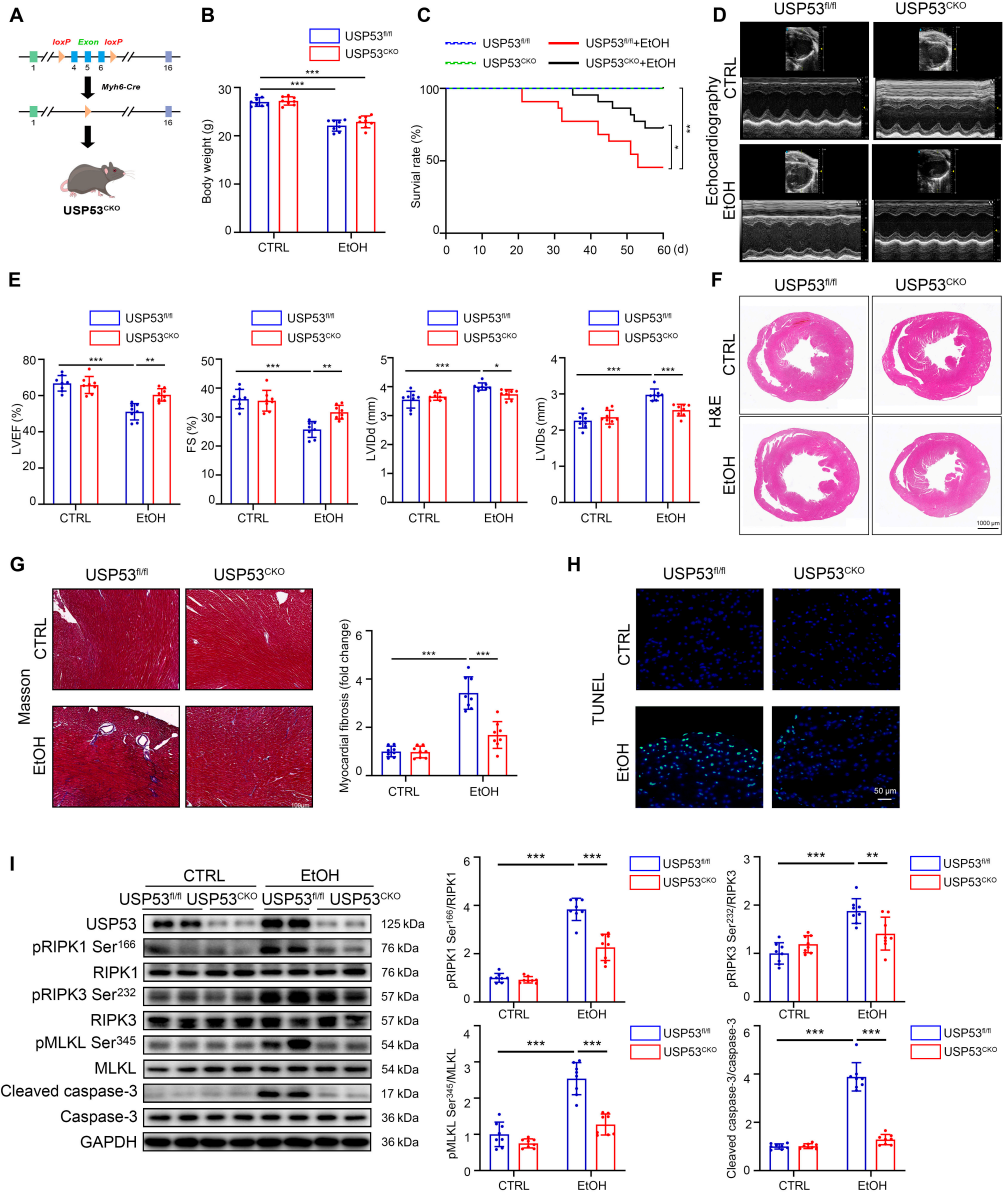
CM-specific USP53 knockout mice develop less alcoholic myocardial injury. (A) Schematic diagram of the generation of USP53^CKO^ mice. (B) Body weights of the mice in the CTRL (USP53^fl/fl^ and USP53^CKO^) and EtOH (USP53^fl/fl^ and USP53^CKO^) groups (*n* = 8 each). (C) Survival curves of the mice in CTRL and EtOH groups. (D and E) Transthoracic echocardiographic results of the LVEF, FS, LVIDd, and LVIDs for cardiac functional analysis (*n* = 8 each). (F) Representative images of heart tissues stained with H&E staining (scale bars, 1,000 μm). (G) Representative images of heart tissues stained with Masson (*n* = 8 each) (scale bars, 100 μm). (H) TUNEL staining of heart tissue of mice from CTRL and EtOH groups (scale bars, 50 μm). (I) Western blotting of pRIPK1 Ser^166^, RIPK1, pRIPK3 Ser^232^, RIPK3, pMLKL Ser^345^, MLKL, cleaved caspase-3, and caspase-3 in heart tissues of the mice in the CTRL and EtOH groups (*n* = 8 each). Results are expressed as the mean ± SD. *P < 0.05, **P < 0.01, ***P < 0.001.

To further investigate the effects of cardiac USP53 in ACM pathogenesis, we generated CM-specific USP53 knockout mice (USP53^CKO^) (Fig. 7A and Fig. S7A). The specificity of USP53 depletion was verified in the heart and other major organs, including lung, liver, kidney, and spleen (Fig. S7B). After feeding with EtOH for 60 d, both USP53^CKO^ and USP53^fl/fl^ mice showed lower body weights than control diet groups (Fig. 7B). Notably, the survival rate of EtOH-fed USP53^CKO^ mice was significantly improved compared with USP53^fl/fl^ mice (Fig. 7C). Meanwhile, echocardiogram indicated that cardiac USP53 deficiency significantly inhibited EtOH-induced cardio-dysfunction in USP53^CKO^ mice, showing as enhanced LVEF and FS, as well as decreased LVIDd and LVIDs (Fig. 7D and E). Consistently, H&E and Masson staining of heart tissue showed significantly improved ventricular chamber dilation and cardiac fibrosis in USP53^CKO^ mice fed with EtOH (Fig. 7F and G and Fig. S7C). Additionally, IHC staining and TUNEL staining demonstrated obviously reduced expression of IL-6 and IL-1β, along with decreased apoptosis level in heart tissue of EtOH-fed USP53^CKO^ mice compared to control mice (Fig. 7H and Fig. S7D). By analyzing the marker of cell death pathways in heart tissue, Western blotting and IHC staining revealed that cardiac USP53 knockout dramatically reversed EtOH-induced up-regulation of pRIPK1/RIPK1, pMLKL/MLKL, and pRIPK3/RIPK3, as well as cleaved caspase-3/caspase-3 (Fig. 7I and Fig. S7E). Together, these findings suggest that CM-specific USP53 deficiency enhances cardiac function and suppresses RIPK1-mediated apoptosis and necroptosis in EtOH-exposed mice.

## Discussion

**Fig. 8.**
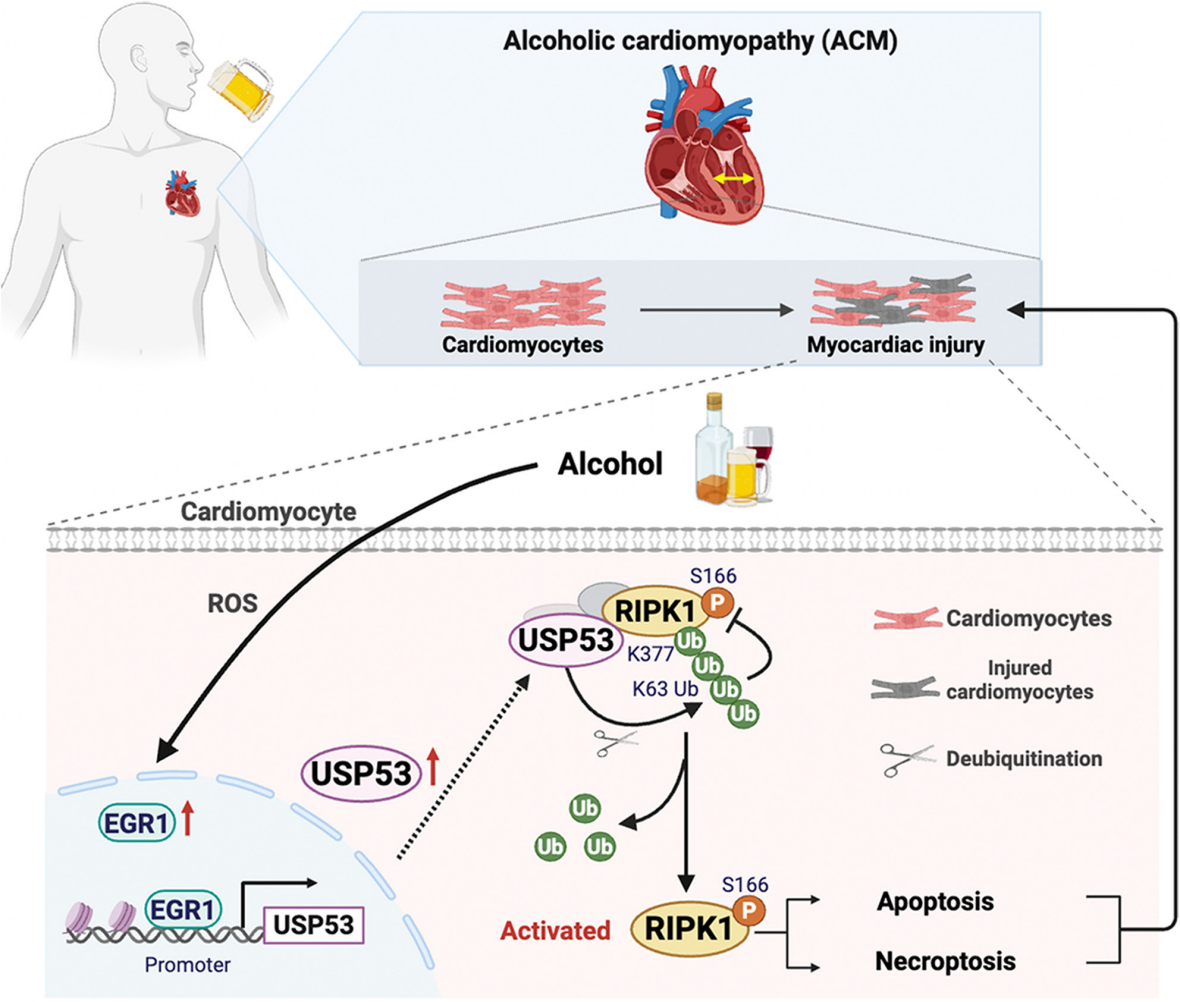
Schematic illustration of USP53-RIPK1 axis in EtOH-exposed CM.

Our study demonstrated the critical role of USP53 in modulating RIPK1 activity and subsequent cell death pathways in ACM. Our major findings revealed that EtOH significantly induced RIPK1-associated apoptosis and necroptosis, which contributed to CM injury and ACM pathogenesis. USP53 served as a crucial regulator of RIPK1 activity by disrupting K63-linked ubiquitination at the K377 site of RIPK1 and facilitating RIPK1 autophosphorylation (Fig. 8). More importantly, our data indicated that CM-specific USP53 knockout rescued against CM apoptosis and necroptosis, as well as cardiac dysfunction of mice fed with EtOH.

CM death plays a pivotal role in the development of ACM. Studies have indicated that alcohol exposure led to multiple types of cell death during ACM development, such as apoptosis, necroptosis, and ferroptosis, in both experimental and clinical models [4,6,9,10]. In order to elucidate the primary form of CM death provoked by EtOH, we assessed key markers of cell death pathways in the chronic EtOH plus interval binge feeding mouse ACM model, which is based on the National Institute on Alcohol Abuse and Alcoholism (NIAAA) models [31]. The chronic EtOH plus binge feeding pattern effectively recapitulates human drinking behaviors, particularly for occasional heavy alcohol intake in modern lifestyles [32]. Our findings indicated that EtOH exposure significantly elevated apoptosis and necroptosis of cardiac cells both in vivo and in vitro. These findings are consistent with previous studies showing that increased apoptosis and necroptosis contribute to CM loss in various EtOH-induced injury models. However, the markers of ferroptosis and pyroptosis were not altered remarkably in the current study. While previous studies reported ferroptosis in the hearts of EtOH-fed mice [9,33], differences in experimental models, such as the age of mice, duration of EtOH exposure, and the pattern of ethanol administration, may account for the discrepancy. These variations suggest that distinct drinking behaviors may activate specific cell death pathways in CMs. Elucidating the intricate network of molecular pathways underlying EtOH abuse and myocardial cell death is essential for unraveling the mechanism of alcoholic cardiac toxicity, as well as offering a potential therapeutic and diagnostic target for ACM.

As a pivotal modulator of cellular survival, inflammatory signaling, and programmed cell death, RIPK1 contains an N-terminal kinase domain, an intermediate region, and a C-terminal death domain [34]. Aberrant RIPK1 kinase activation contributes significantly to myocardial injury. Research has proven that RIPK1 activation exacerbated diabetic cardiac injury and dilated cardiomyopathy [18,35]. Recent findings also indicated that RIPK1-dependent necroptosis can exacerbate alcohol-induced myocardial injury [7,36]. Our study revealed that alcohol treatment triggered the concurrent activation of necroptosis and apoptosis pathways in vivo and in vitro, which is accompanied by the elevation of RIPK1 autophosphorylation at S166. This observation indicates that EtOH may induce dual cell death pathways via RIPK1 kinase activation and pRIPK1-dependent signaling. This hypothesis was further supported by the attenuation of apoptosis and necroptosis upon Nec-1 treatment, a selective RIPK1 kinase inhibitor [37]. Given that both necroptosis and apoptosis share upstream pathways and are regulated by RIPK1 kinase activity, the simultaneous induction of RIPK1-dependent apoptosis and necroptosis is plausible. This concept is further supported by studies demonstrating that Nec-1 effectively suppresses both forms of cell death in various pathological contexts, including brain ischemia, lung ischemia–reperfusion injury, and osteoarthritis [38–40]. Thus, our findings highlight RIPK1 as a promising therapeutic target for ACM. Deciphering the mechanisms of alcohol-induced RIPK1 activation will provide critical insights into ACM pathogenesis.

As a reversible posttranslational modification, protein ubiquitination is catalyzed by E3 ubiquitin ligases and counteracted by deubiquitinating enzymes, playing key roles in controlling protein function or stability [41]. Ubiquitination is a critical and highly regulated posttranslational modification of RIPK1 [42]. While we observed decreased RIPK1 ubiquitination in EtOH-treated CMs, the expression of known RIPK1 ubiquitination regulators (CYLD, cIAP1, and cIAP2) remained unchanged. To unveil the molecular mechanisms underlying the regulation of RIPK1 in ACM, we identified USP53 as a binding partner of RIPK1 through Co-IP coupled with an LC-MS/MS assay. USP53 has been demonstrated to play critical roles in diverse cellular processes, such as in nervous system injury [43], osteogenic differentiation [44–46], DNA damage repair [47], and tumorigenesis [22–25]. Although USP53 was initially thought to be catalytically inactive due to a missing histidine residue in its catalytic pocket [48], emerging evidence indicates that it functions as a deubiquitinase with preferential selectivity for K63-linked polyubiquitin chains [49]. However, whether USP53 influences RIPK1 activity, particularly pRIPK1-driven apoptosis and necroptosis, in ACM has yet to be investigated. In the current study, we demonstrate for the first time that USP53 expression is up-regulated in CMs following alcohol exposure. Specific knockout of USP53 in CMs inhibited RIPK1-dependent apoptosis and necroptosis. Mechanistically, USP53 directly interacts with RIPK1 and activates RIPK1 kinase activity by disrupting K63-linked ubiquitination at K377, the major K63 ubiquitination site in human RIPK1 [50]. Notably, K63-linked ubiquitination at K377 of RIPK1 is closely associated with RIPK1 kinase activation. Previous studies indicate that mice expressing RIPK1 K376R (corresponding to the human RIPK1 K377R), which is defective in RIPK1 ubiquitination, die during embryogenesis. Mechanistically, cells expressing RIPK1 K376R are more susceptible to TNF-α-induced apoptosis and necroptosis with increased RIPK1 activation, while the deletion of caspase-8 and RIPK3 prevents embryonic lethality in mice [17,50]. It is noteworthy that our study also observed similar phenomena. The K377R mutation can lead to increased RIPK1 autophosphorylation under alcohol stimulation, accompanied by aggravated cell apoptosis. Meanwhile, we found that overexpression of USP53 did not abolish ubiquitination of RIPK1 K377R mutation, nor could it significantly increase apoptosis. These results provide robust evidence for the functional significance of deubiquitination at K377 in RIPK1-dependent cell death pathway. Additionally, after knocking down USP53, overexpression of USP53 can once again promote the RIPK1-dependent death phenotype, but overexpression of USP53-C41S fails to re-activate RIPK1-dependent necroptosis and apoptosis. These findings collectively support the model in which USP53-mediated deubiquitination at K377 promotes RIPK1 activation and contributes to EtOH-induced CM injury. Targeting the USP53–RIPK1 axis could be a potential strategy for the treatment of alcohol-associated myocardial damage.

The molecular mechanisms driving EtOH-mediated USP53 up-regulation remain unclear. ROS are considered a phenotype significantly associated with EtOH [51]. We verified that ROS production was indeed increased by EtOH treatment in CMs, both in vivo and in vitro, under our experimental conditions. Song et al. [52] discovered that EtOH can enhance the expression of NOX4 by activating the AngII-AT1R signaling pathway, leading to increased production of ROS and subsequent CM apoptosis. In addition, Qian et al. [6] found that AdipoRon ameliorated chronic EtOH-induced cardiac necroptosis by reducing ceramide de novo synthesis and ROS. These findings suggest that ROS is a major mediator that can directly damage CMs and indirectly activate intracellular signaling pathways, exacerbating cellular injury. In addition, our study identified EGR1 as a transcriptional regulator of USP53 through TF prediction and further in vitro validation. We found that alcohol exposure increased EGR1 expression, and knockdown of EGR1 markedly reduced USP53 level. These results align with prior reports demonstrating that EtOH enhances EGR1 expression and nuclear translocation, which have been implicated in inhibiting osteogenic differentiation of bone marrow mesenchymal stem cells and promoting chronic EtOH-induced hepatic steatosis [53,54]. Given that EGR1 has been reported to be up-regulated by ROS [55,56], we found that pretreatment with the ROS inhibitor NAC attenuated EtOH-induced up-regulation of both EGR1 and USP53, thereby suppressing RIPK1-dependent apoptosis and necroptosis. Notably, the protective effects of NAC and EGR1 knockdown were abolished by USP53 overexpression. These results indicated that ROS induced by EtOH promoted EGR1–USP53–RIPK1 axis and RIPK1-dependent apoptosis and necroptosis in CMs.

In summary, our study reveals that alcohol exacerbates cardiac injury by promoting RIPK1-dependent apoptosis and necroptosis, while USP53 is a critical regulator of RIPK1 activation by preferentially deubiquitinating the K63-linked ubiquitin chain. We showed that targeted deletion of USP53 in CMs protects against EtOH-induced cardiac dysfunction by blocking RIPK1-dependent apoptotic and necroptotic pathways. Our work elucidates the pathogenic role of the USP53–RIPK1 signaling axis in EtOH-induced cardiomyopathy, providing a novel molecular target for therapeutic intervention.

## Methods

### Animals

Adult C57BL/6J mice (8 weeks old; male) were purchased from Shanghai Model Organisms Center Inc. The mice were maintained at 23 ± 3 °C and 30% to 70% humidity, with 12-h light/12-h dark circulation. The study was approved by the Ethical Committee of School of Basic Medical Science of Shandong University (ECSBMSSDU2024-2-21) and the Experimental Animal Ethics Committee of Qilu Hospital of Shandong University (DWLL-2024-419). We constructed the ACM mouse model using the Lieber–DeCarli liquid diet and intragastric alcohol administration method recommended by the NIAAA [31]. In brief, mice in the EtOH group were provided with the Lieber–DeCarli liquid diet (5% v/v EtOH) for 60 d, along with intragastric EtOH administration (5 g/kg) every 10 d. Meanwhile, mice in the CTRL group were provided with the control Lieber–DeCarli liquid diet for 60 d, along with intragastric maltose dextrin administration (9 g/kg) every 10 d. To examine the effect of Nec-1 on ACM, we grouped the mice according to the following strategy: (a) CTRL + an equivalent dose of dimethyl sulfoxide (DMSO); (b) EtOH + an equivalent dose of DMSO; (c) EtOH + Nec-1 (1.8 mg/kg/d).

To further investigate the effects of cardiac USP53 in ACM pathogenesis, we generated CM-specific USP53 knockout mice. Subsequently, we grouped the mice according to the following strategy: (a) CTRL (USP53^fl/fl^); (b) CTRL (USP53^CKO^); (c) EtOH (USP53^fl/fl^); (d) EtOH (USP53^CKO^).

### Echocardiography

A Vevo 2100 system (VisualSonics, Toronto, ON, Canada) was used to acquire transthoracic echocardiograms, with mice anesthetized using isoflurane and positioned on a heating pad. M-mode recording was performed from the parasternal long-axis view. Data on the average LVIDs and LVIDd, as well as the LVEF and FS, were collected from 3 consecutive heart cycles.

### Histopathology

Heart tissues were fixed, paraffin-embedded, and sectioned at a thickness of 5 μm. Sections were dewaxed, rehydrated, and processed according to standard histological protocols. H&E staining was used to assess cardiac morphology, while collagen deposition was evaluated by Masson staining using a commercial kit (Solarbio, catalog no. G1346).

### DHE staining

The heart tissues were fixed with formalin and cut into 5-μm-thick paraffin sections. The sections were then dewaxed and stained with a DHE assay kit (Beyotime, catalog no. S0063). The slices were subsequently viewed under a microscope to evaluate oxidative stress levels in the heart tissues of mice.

### Immunohistochemistry

Heart tissues were fixed, paraffin-embedded, and sectioned at a thickness of 5 μm. Antigen retrieval was carried out using a sodium citrate buffer after deparaffinization and rehydration, and 0.3% hydrogen peroxide was used to block endogenous peroxidase activity. After blocking for an hour to avoid nonspecific binding, they were incubated overnight at 4 °C with the primary antibody. Primary antibodies used included USP53 (Immunoway, YT4845), cleaved caspase-3 [Cell Signaling Technology (CST), 9661T], p-MLKL (Ser^345^, Abcam, ab196436), IL-6 (Abcam, ab290735), and IL-1β (Abcam, ab315084). Immunostaining was carried out using a commercial detection kit (Zhongshan Golden Bridge Biotechnology, catalog no. PV-9000).

### Isolation of CMs and CFs

Primary CMs and CFs were isolated from 3-d-old neonatal Sprague–Dawley rats. The specific operational procedures are as previously described [57]. In summary, excised hearts were minced and enzymatically dissociated using type II collagenase. The resulting cell suspension was filtered and subjected to differential adhesion in an incubator. After 1.5 h, the adherent cells, identified as CFs, were cultured in Dulbecco’s modified Eagle’s medium (DMEM) supplemented with 10% fetal bovine serum (FBS) for 24 h. The nonadherent CMs were collected from the supernatant, and viable cells were counted. CMs were then seeded and maintained in DMEM containing 10% calf serum and 0.1 mM 5-bromo-2′-deoxyuridine for 48 h prior to medium replacement and subsequent experimental treatments.

### Cell culture and drug treatment

The primary CMs and CFs were extracted and cultured using the aforementioned method. AC16 cells were cultured in DMEM/F12 (enriched with 10% FBS). Subsequently, the cells were exposed to 200 mM EtOH or phosphate-buffered saline (PBS) for 24 h. HEK-293T cells were cultured in DMEM (enriched with 10% FBS). The siRNA and plasmids were synthesized from the GenePharma Company (Shanghai GenePharma, China). USP53-siRNA: GUGCGGUACAUUUCUACAATT; UUGUAGAAAUGUACCGCACTT. EGR1-siRNA: ACCCUAAGCUGGAGGAGAUTT; AUCUCCUCCAGCUUAGGGUTT. Cell transfection was performed using Lipofectamine 2000 (Thermo Fisher Scientific, catalog no. 11668019).

For additional administration, Z-VAD-FMK (30 μM, Glpbio, catalog no. GC12861), Fer-1 (10 μM, Glpbio, catalog no. GC10380), Nec-1 (30 μM, Glpbio, catalog no. GC11008), and NAC (5 μM, MedChemExpress, catalog no. HY-B0215) were applied to the medium 1 h before EtOH.

### Cell viability assay

Cell viability was assessed using a CCK8 assay kit (Glpbio, catalog no. GK10001). Briefly, AC16 cells were seeded into 96-well plates and allowed to adhere for 24 h. Following treatment, 10 μl of CCK8 solution was added to 90 μl of culture medium in each well. After a 2-h incubation at 37 °C, absorbance was measured at 450 nm using a microplate reader to evaluate cell viability.

### Western blotting analysis

Cells and heart tissues were lysed using radioimmunoprecipitation assay (RIPA) buffer (Solarbio, catalog no. R0010) supplemented with 1% phenylmethanesulfonyl fluoride (PMSF; Solarbio, catalog no. FJP0100) and 1% phosphatase inhibitor cocktail (BOSTER, catalog no. AR1183). Protein concentrations were quantified using a bicinchoninic acid (BCA) protein assay kit (Thermo Fisher Scientific, catalog no. 23225). Equal amounts of protein were resolved by 10% or 12% sodium dodecyl sulfate–polyacrylamide gel electrophoresis (SDS–PAGE) and transferred onto polyvinylidene difluoride (PVDF) membranes. Membranes were blocked in blocking solution (BOSTER, catalog no. AR0041) for 1 h at room temperature, followed by overnight incubation at 4 °C with the appropriate primary antibodies.

After washing, membranes were incubated with horseradish peroxidase-conjugated secondary antibodies for 1 h at room temperature. Protein bands were visualized using enhanced chemiluminescence (Millipore, catalog no. WBKLS0500) and imaged using the Amersham ImageQuant 680 system (GE Healthcare, Boston, USA). Band intensities were quantified with ImageJ software, and protein expression levels were normalized to GAPDH and expressed as a percentage relative to the control group. All experiments were independently repeated at least 3 times.

Primary antibodies used included USP53 (Immunoway, YT4845), p-RIPK1 (Ser^166^; Proteintech, 28252-1-AP), RIPK1 (CST, 3493T), p-RIPK3 (Ser^227^, Abcam, ab209384; Ser^232^, Abcam, ab195117), RIPK3 (CST, 10188T), p-MLKL (Ser^358^, Abcam, ab187091; Ser^345^, Abcam, ab196436), MLKL (Immunoway, YM8455), cleaved caspase-3 (CST, 9661T), caspase-3 (CST, 9662T), EGR1 (CST, 4153T), ubiquitin (CST, 20326T), K63-linked ubiquitin (CST, 5621T), K48-linked ubiquitin (CST, 8081T), HA (CST, 3724T), Flag (CST, 14793T), MYC (CST, 2276T), cleaved GSDMD (Abcam, ab215203), GSDMD (Proteintech, 20770-1-AP), ACSL4 (Proteintech, 22401-1-AP), GPX4 (CST, 59735T), and glyceraldehyde-3-phosphate dehydrogenase (GAPDH) (Proteintech, 60004-1-AP).

### Immunofluorescence staining

Cells were seeded onto glass slides and fixed with 4% paraformaldehyde. After fixation, the cells were incubated overnight at 4 °C with the indicated primary antibodies. The following day, fluorescently labeled secondary antibodies were applied for 2 h at room temperature. Nuclei were counterstained, and the slides were mounted using an anti-fade mounting medium containing 4′,6-diamidino-2-phenylindole (DAPI). Fluorescence signals were visualized and captured using a fluorescence microscope.

### TUNEL staining

In this study, TUNEL assays were performed using a TUNEL BrightGreen Apoptosis Detection Kit (Vazyme, catalog no. A112). At the conclusion of staining, the nuclei were labeled with DAPI, and TUNEL-positive cells were observed under a microscope to evaluate the extent of cell apoptosis. The percentage of TUNEL-positive cells was determined by the ratio of TUNEL-positive cells to DAPI-positive nuclei.

### Transmission electron microscopy

Cultured AC16 cells, with or without stimulation, were collected and fixed in a solution containing 2.5% glutaraldehyde and 2% paraformaldehyde in 0.1 M sodium cacodylate buffer (pH 7.4) at 4 °C for at least 2 h. After fixation, the samples were rinsed with 0.1 M sodium cacodylate buffer and postfixed with 1% osmium tetroxide (OsO₄) and 1.5% potassium ferrocyanide [K₄Fe(CN)₆] for 1 h. The cells were then washed with distilled water and stained with 1% aqueous uranyl acetate for 1 h, followed by additional water washes and a graded EtOH dehydration series.

Subsequently, samples were treated with propylene oxide for 1 h and infiltrated overnight in a 1:1 mixture of propylene oxide and TAAB Epon resin (Marivac Canada Inc., St. Laurent, Canada). After infiltration, the specimens were embedded in pure TAAB Epon and polymerized at 60 °C for 48 h. Ultrathin sections (~60 nm) were obtained using a Reichert Ultracut-S ultramicrotome, mounted on copper grids, counterstained with lead citrate, and examined using a JEOL 1400 or Tecnai G2 Spirit BioTWIN TEM.

### Co-IP assay

The cells were collected in NP-40 lysis buffer (BOSTER, catalog no. AR0107) containing 1% PMSF and 1% phosphatase inhibitors. The 10% of the cell lysate was used as an input control. The rest of the cell lysate was incubated with control immunoglobulin G (IgG) or specified IP primary antibodies (1:50 dilution) and Protein A/G Magnetic Beads (Selleck, catalog no. B23202) on the rotary agitation overnight at 4 °C. After incubation, the beads were washed 3 times. The antigen–antibody complexes were eluted from the beads, a loading buffer was added, and the samples were boiled in a metal bath at 95 °C for 5 min. Then, silver staining (Beyotime, catalog no. P0017S), LC-MS/MS, and Western blotting analyses were performed.

### Ubiquitination assay

The ubiquitination assay was conducted as previously described [58]. Briefly, cells were lysed in NP-40 lysis buffer containing 1% PMSF and 1% phosphatase inhibitors. The protein concentration was determined to ensure equal protein loading. Ten percent of the cells were lysed in loading buffer for the input, and the remaining supernatants were incubated with IP primary antibodies (diluted 1:50) and Protein A/G Magnetic Beads on a rotary agitator overnight at 4 °C. Following extensive washing the next day, loading buffer was added, and the samples were boiled in a metal bath at 95 °C for 5 min, followed by Western blotting analysis.

For the endogenous study, AC16 cells were transfected with either small interfering negative control RNA or USP53-siRNA for 48 h, and cell lysate was subjected to subsequential study. For exogenous study, HEK-293T cells were transfected with relevant plasmids for 48 h and the cells were treated with 10 μM MG132 (Glpbio, catalog no. GC10383) for an additional 6 h before harvesting.

### Reverse transcription quantitative polymerase chain reaction assay

The RNA-easy Isolation Reagent (Vazyme, catalog no. R711) was utilized to extract total RNA, which was subsequently converted into cDNA using the HiScript III RT SuperMix for quantitative polymerase chain reaction (qPCR) (Vazyme, catalog no. R323-01). Finally, qPCR was performed using ChamQ SYBR qPCR Master Mix (Vazyme, catalog no. Q711-02) on a CFX96 Touch Real-Time PCR System (Bio-Rad, Hercules, CA, USA). The PCR primers were designed as follows: human USP53-F: CTCAGGAATTTGGAAGCAGGC, human USP53-R: TCTTGCATTATGGTCCTTGGGT; human RIPK1-F: TAAGAAGAATGGCGGCACCC, human RIPK1-R: TTCTCTGTGGGCTTTGCGTT; human EGR1-F: CCCCGACTACCTGTTTCCAC, human EGR1-R: TGGGTTTGATGAGCTGGGAC; human GAPDH-F: GCACCGTCAAGGCTGAGAAC, human GAPDH-R: TGGTGAAGACGCCAGTGGA.

### Detection of intracellular ROS generation

ROS generation was investigated by DCFH-DA assay (Beyotime, catalog no. S0033S). Briefly, AC16 cells were randomly seeded in a 24-well plate at a density of 1 × 10^5^ cells/well for 12 h before further manipulation. The groups were divided as follows: (a) control; (b) control + NAC (5 μM); (c) EtOH; and (d) EtOH + NAC (5 μM). Then, the cells were cultured with DCFH-DA (10 μM, diluted with DMEM/F12 medium) for 20 min in a 37 °C incubator. After rinsing with PBS for 3 times, DCFH-DA fluorescence images of AC16 cells were obtained by fluorescence microscope.

### Statistical analysis

All data were analyzed using Prism 8.0 (GraphPad). Unpaired 2-tailed Student’s *t* tests were used to compare the 2 groups. One-way analysis of variance (ANOVA) and Tukey’s honestly significant difference (HSD) tests were used to compare multiple samples. Survival curves were compared using log-rank tests. All experiments were repeated no fewer than 3 times, and the data are expressed as means ± standard deviation (SD). *P* < 0.05 was considered to indicate statistical significance.

## Data Availability

The data that support the findings of this study are available from the corresponding author upon reasonable request.
