# Supplementary material for: USP53 Drives Ethanol-Induced Myocardial Injury by Promoting K63 Deubiquitination-Dependent RIPK1 Activation at K377
Source: Research (Wash D C). 2025 Aug 14;8:0823. doi: 10.34133/research.0823 (PMC12352791; doi:10.34133/research.0823)
Supplement: Supplementary 1 — Figs. S1 to S7 [file research.0823.f1.docx]

**Supplementary Material**

**USP53 Drives ethanol-induced myocardial injury by Promoting K63 Deubiquitination-dependent RIPK1 Activation at K377**

Jichen Pan^1, #^, Xiaolin Liu^1, #^, Xiao Li^1^, Shanshan Wang^2^, Yuliang Zhao^2^，Chong Yuan^1^, Dongdong Liu^1^, Liyan Wang^3^, Meng Zhang^1^, Fengming Liu^2, *^, Mei Zhang^1,*^, and Shen Dai^2, *^

^1^ State Key Laboratory for Innovation and Transformation of Luobing Theory; Key Laboratory of Cardiovascular Remodeling and Function Research of MOE, NHC, CAMS and Shandong Province; Department of Cardiology, Qilu Hospital of Shandong University, Jinan 250012, China

^2^ School of Basic Medical Sciences, Shandong University, Jinan250012, China

^3^ Morphological Experimental Center, School of Basic Medical Sciences, Shandong University, Jinan 250012, China

^#^ These authors contributed to the work equally and should be regarded as co-first authors

^*^Corresponding Authors

**Address correspondence to**: [fengmingliu@sdu.edu.cn](mailto:fengmingliu@sdu.edu.cn) (Fengming Liu), [daixh@vip.sina.com](mailto:daixh@vip.sina.com) (Mei Zhang), and [shen.dai@sdu.edu.cn](mailto:shen.dai@sdu.edu.cn) (Shen Dai)


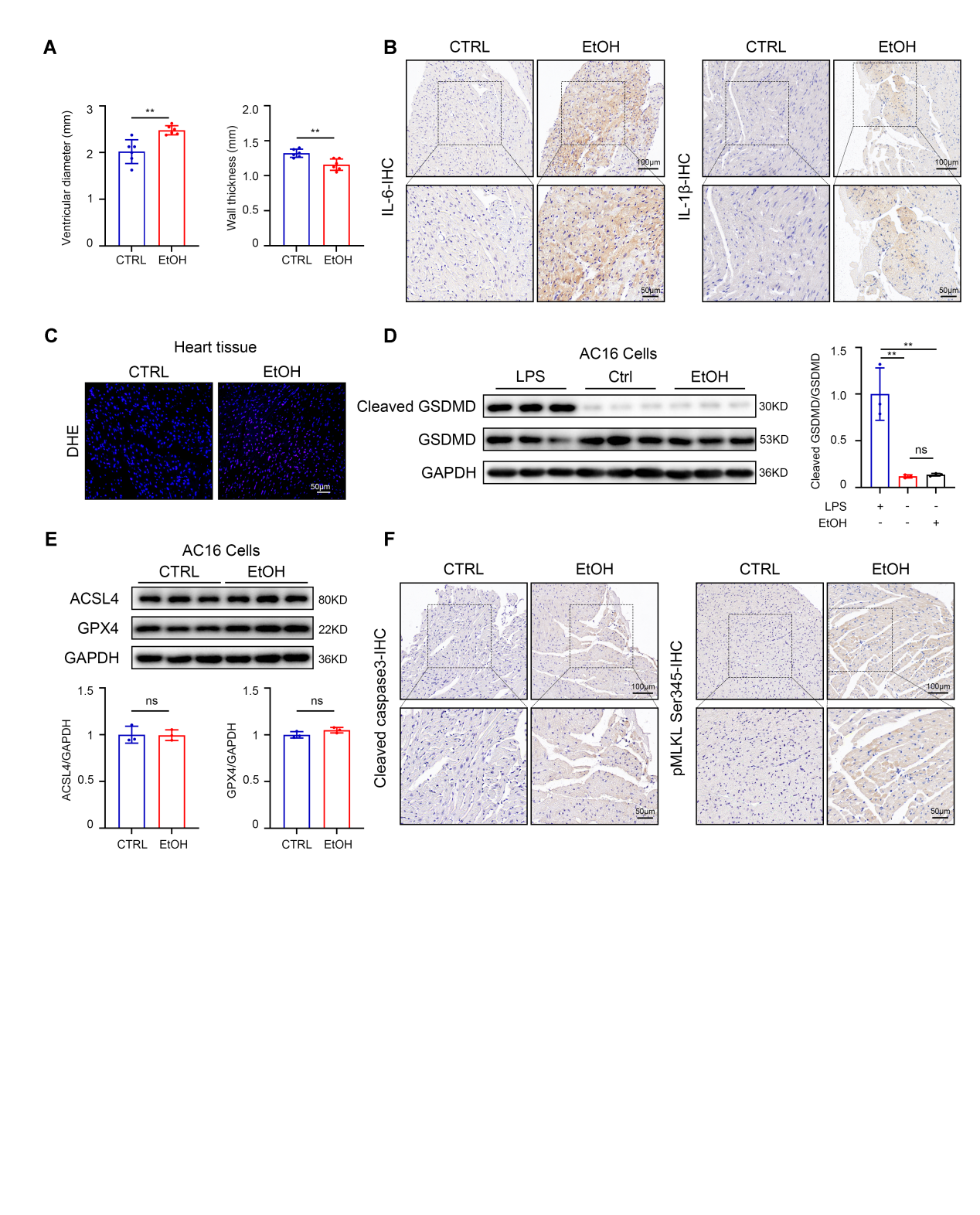


Figure S1

(A) Quantification of ventricular wall thickness and cardiac chamber dimensions of hearts from alcohol-fed and control mice (n=6 each); (B) Representative images of immunohistochemistry (IHC) staining showing IL-6 and IL-1β in the heart tissue from ethanol-exposed mice and control (Scale bars, 100μm; 50μm); (C) Images of superoxide anion generation in heart tissues of ACM mice models detected by dihydroethidium (DHE) fluorescence probe (Scale bars, 50μm); (D) Western blotting of Cleaved GSDMD and GSDMD in ethanol-treated AC16 cells (n=3 each), LPS treatment (1ug/ml) was used as control; (E) Western blotting of ACSL4 and GPX4 in ethanol-treated AC16 cells (n=3 each). (F) IHC staining showing pMLKL and Cleaved caspase3 in the heart tissue from ethanol-exposed mice and control (Scale bars, 100μm; 50μm). Results are expressed as the mean±SD; *P<0.05, **P<0.01, ***P<0.001.


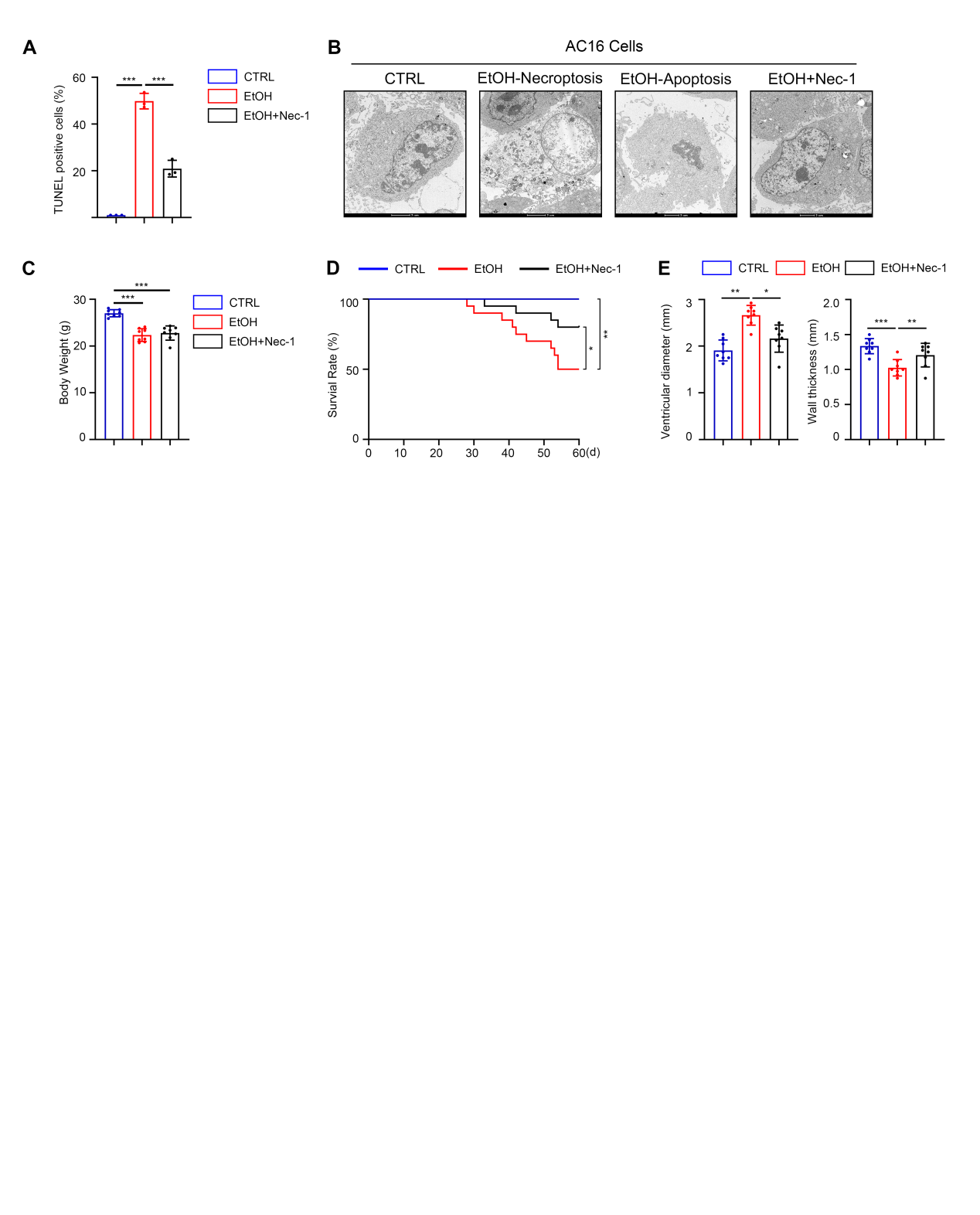


Figure S2

(A) Analysis of TUNEL in the CTRL, EtOH and EtOH+Nec-1 groups of AC16 cells (n=3 each); (B) Transmission electron microscopy assay in the CTRL, EtOH and EtOH+Nec-1 groups of AC16 cells; (C) Body weight of the mice in CTRL, EtOH, and EtOH+Nec-1 groups (n=8 each); (D) Survival rate of the mice in CTRL, EtOH, and EtOH+Nec-1 groups; (E) Quantification of ventricular wall thickness and cardiac chamber dimensions of hearts from mice fed with alcohol, with or without Nec-1 treatment (n=8 each). Results are expressed as the mean±SD; *P<0.05, **P<0.01, ***P<0.001.


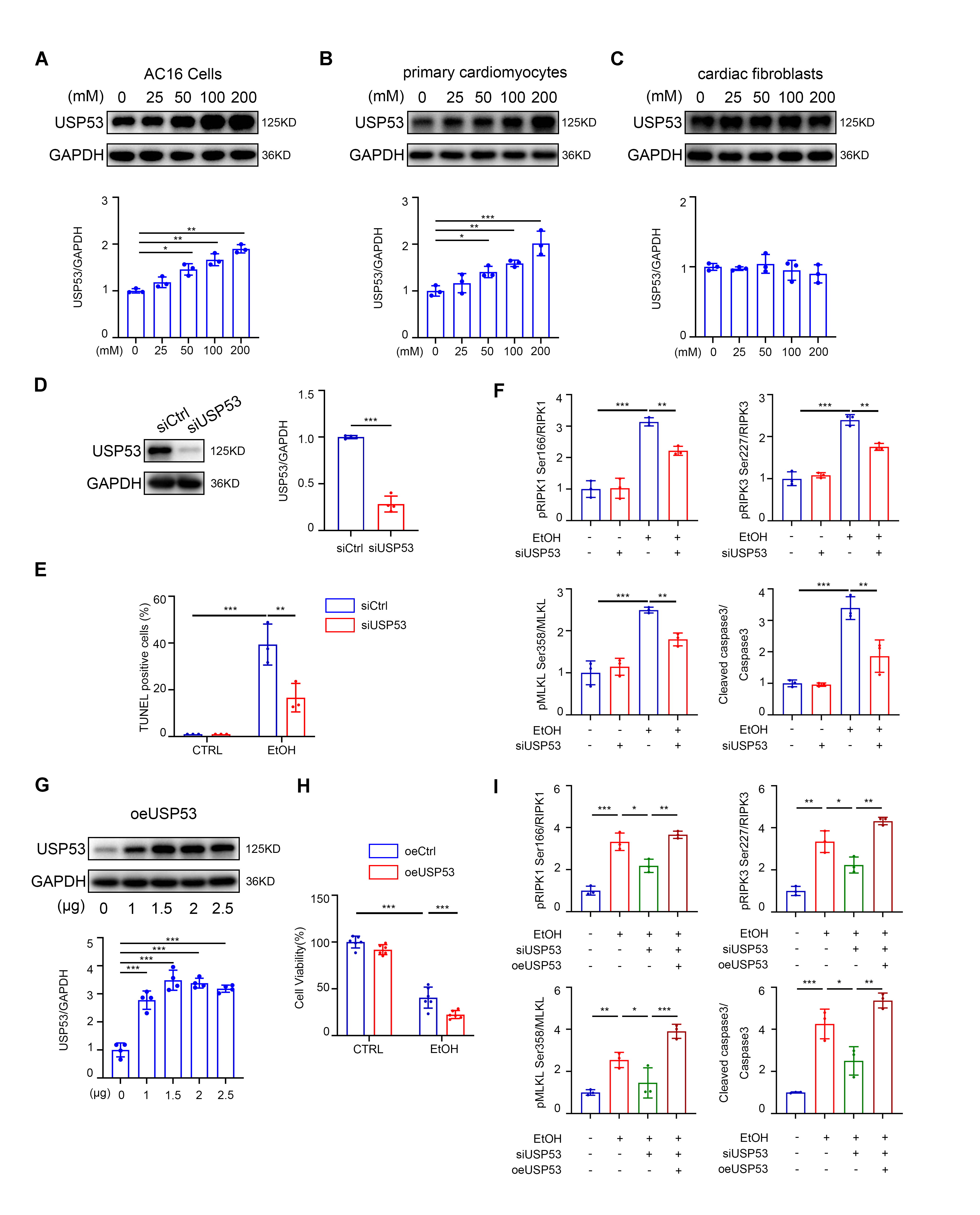


Figure S3

(A)-(C) Western blotting of USP53 in gradient ethanol-treated AC16 cells, primary cardiomyocytes and cardiac fibroblasts (n=3 each); (D) Western blotting of USP53 in the AC16 cells transfected with USP53 siRNA (n=3 each); (E) Analysis of TUNEL in the CTRL+siCtrl, CTRL+siUSP53, EtOH+siCtrl, and EtOH+siUSP53 groups of AC16 cells (n=3 each); (F) Quantification of pRIPK1 Ser166, pRIPK3 Ser227, pMLKL Ser358, and Cleaved caspase3 levels in ethanol-exposed AC16 cells by western blotting (n=3 each); (G) Western blotting of USP53 in the AC16 cells transfected with increasing amounts of USP53 overexpression plasmid (n=4 each); (H) CCK8 assays of AC16 cells transfected with USP53 overexpression plasmid under ethanol treatment (n=6 each). Results are expressed as the mean±SD; *P<0.05, **P<0.01, ***P<0.001.


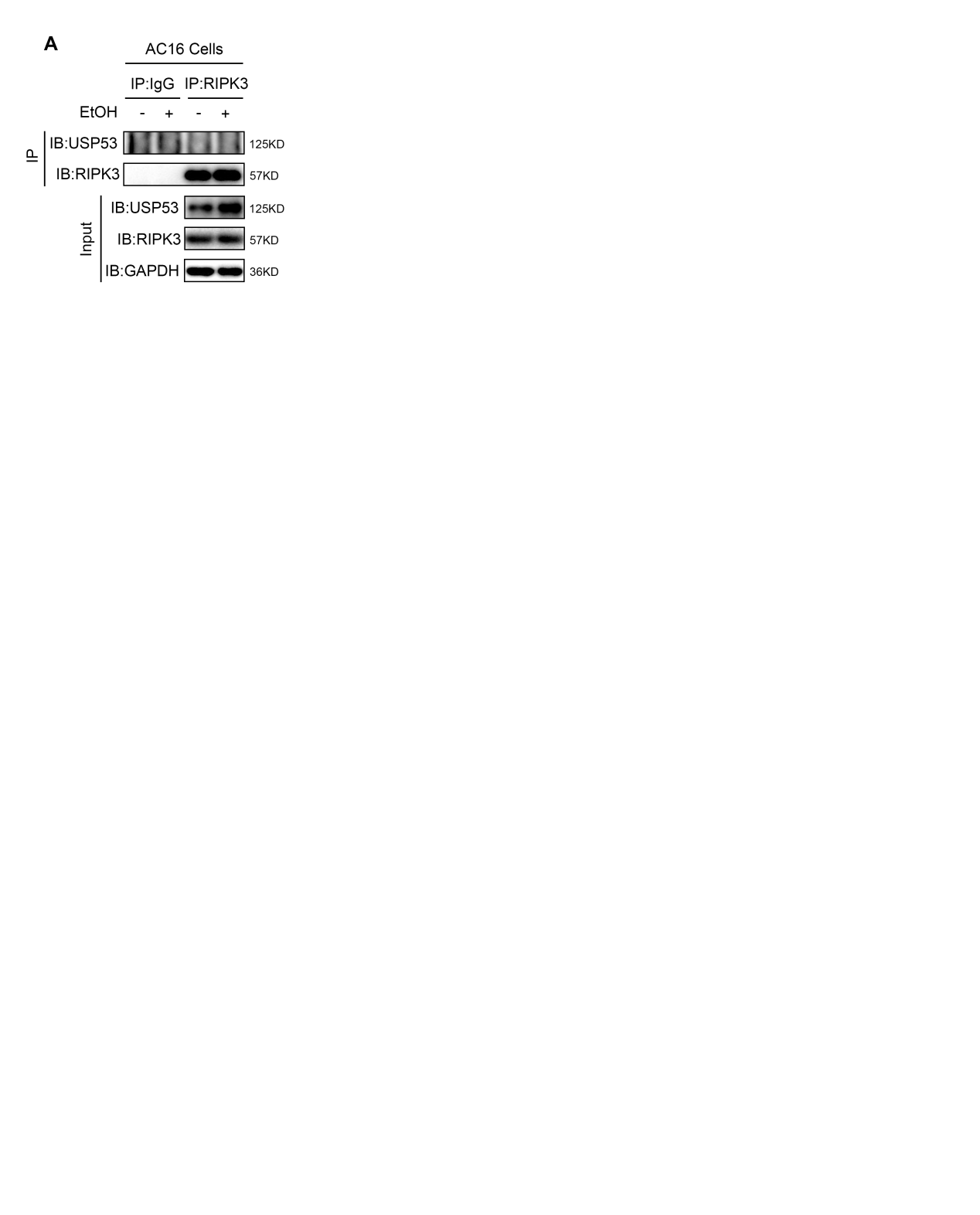


Figure S4

(A) Co-immunoprecipitation (Co-IP) assay of USP53 and RIPK3 in AC16 cells with or without ethanol treatment.


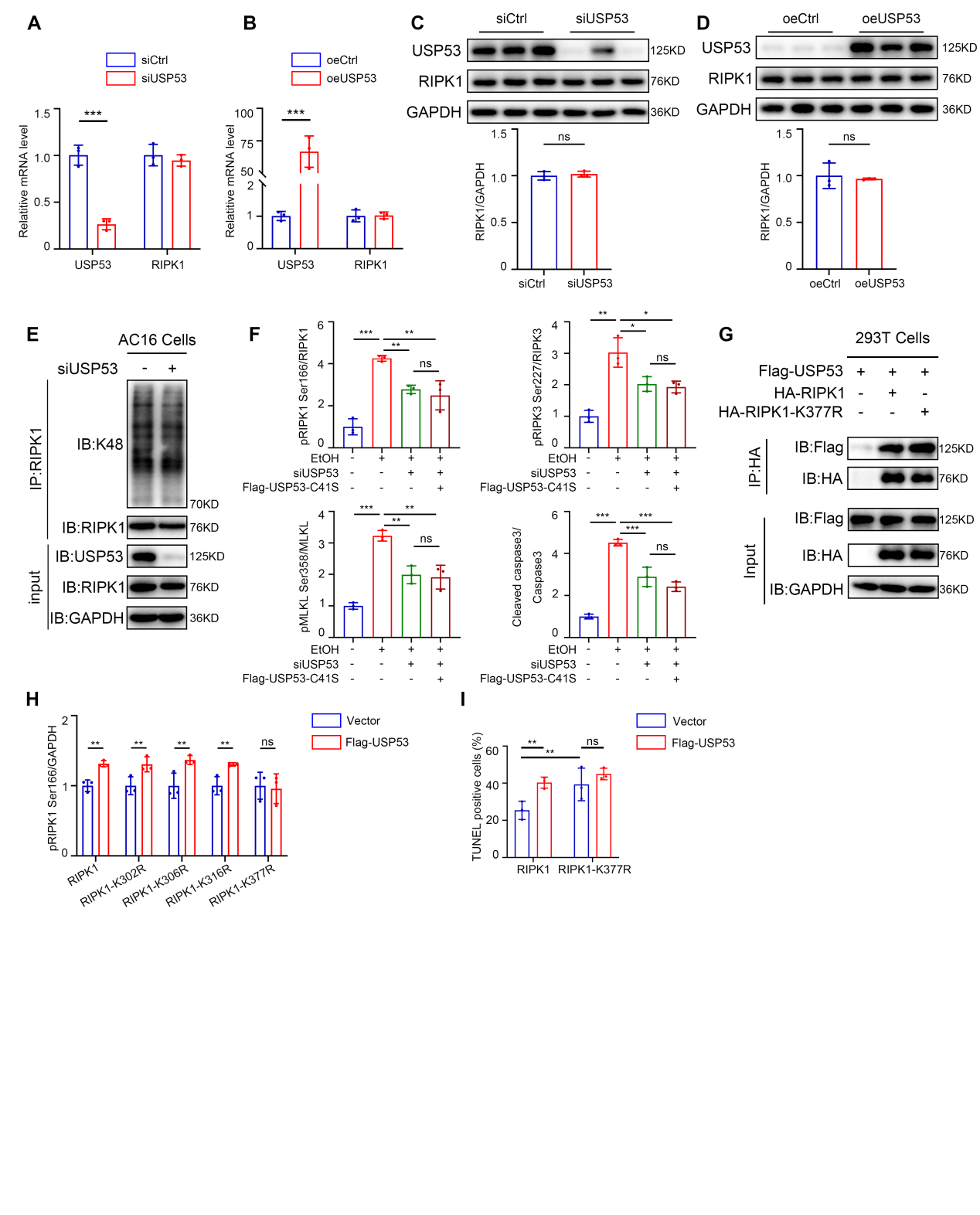


Figure S5

(A) and (B) PCR analysis of USP53 and RIPK1 expression in the AC16 cells with USP53 knockdown or overexpression (n=3 each); (C) and (D) Western blotting of USP53 and RIPK1 in the AC16 cells with USP53 knockdown or overexpression (n=3 each); (E) K48 ubiquitination of immunoprecipitated RIPK1 in AC16 cells transfected with USP53 siRNA; (F) Quantification of western blotting results showing pRIPK1 Ser166, pRIPK3 Ser227, pMLKL Ser358, and Cleaved caspase3 levels in USP53-knockdown AC16 cells with ethanol exposure, with or without USP53-C41S mutant reconstitution (n=3 each); (G) Co-immunoprecipitation (Co-IP) assay of H293T cells co-transfected with Flag-USP53 and either HA-RIPK1 or HA-RIPK1-K377R mutant; (H) Quantification of western blotting results showing pRIPK1 Ser166 level in the ethanol-treated AC16 cells with USP53 or RIPK1-mutant overexpression (n=3 each); (I) Quantification of TUNEL staining showing ethanol-stimulated AC16 cells co-transfected with Flag-USP53 and RIPK1 or RIPK1-K377R mutant (n=3 each). Results are expressed as the mean±SD; *P<0.05, **P<0.01, ***P<0.001.


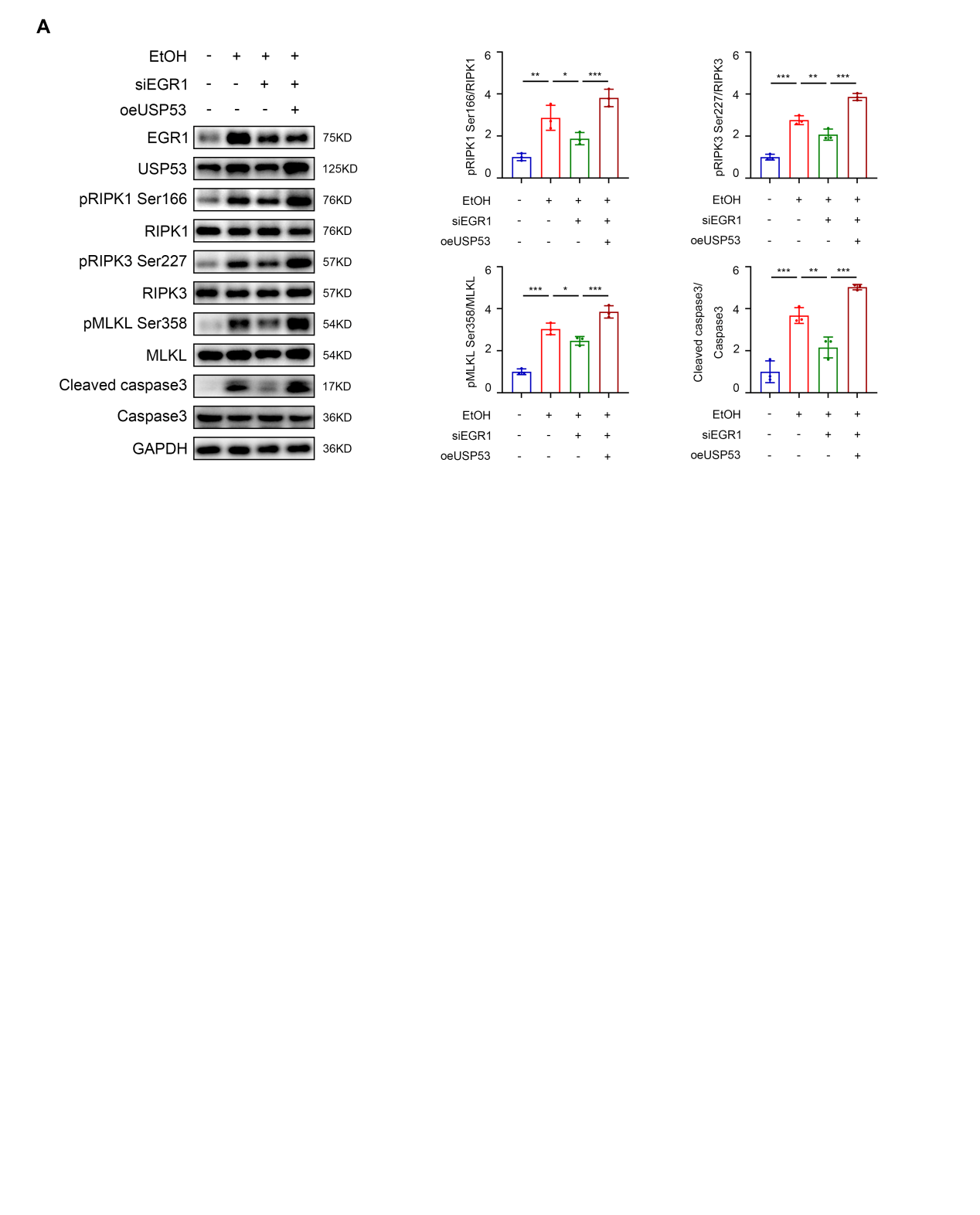


Figure S6

(A) Western blotting and quantification analysis of pRIPK1 Ser166, pRIPK3 Ser227, pMLKL Ser358, and Cleaved caspase3 levels in EGR1 knockdown AC16 cells with ethanol treatment, with or without USP53 overexpression (n=3 each). Results are expressed as the mean±SD; *P<0.05, **P<0.01, ***P<0.001.


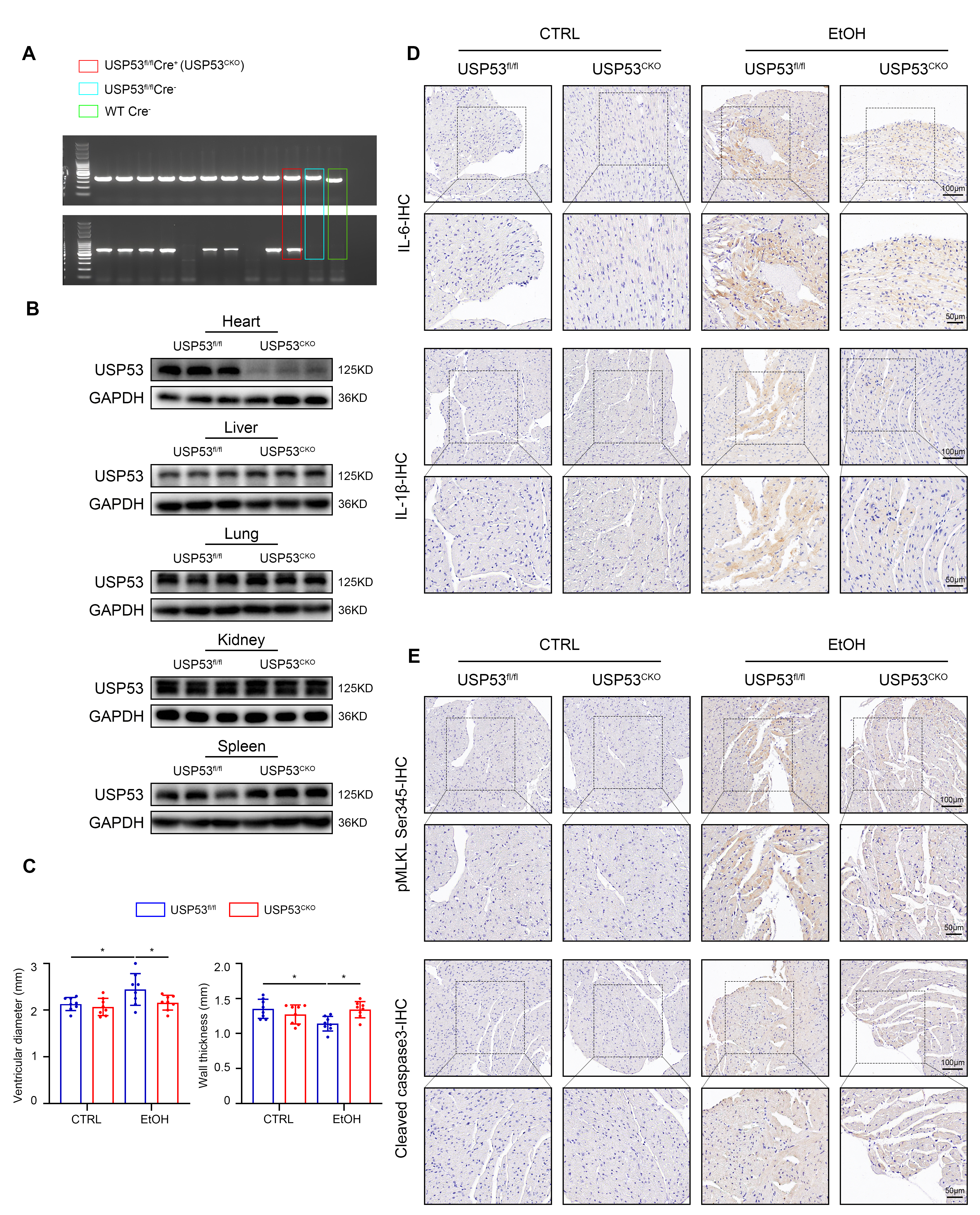


Figure S7

(A) Genotype of cardiomyocyte-specific USP53 knockout mice; (B) Western blotting of USP53 in the heart, liver, spleen, lung and kidney of cardiomyocyte-specific USP53 knockout mice. (C) Quantification of ventricular wall thickness and cardiac chamber dimensions of hearts from alcohol-fed USP53^CKO^ mice and control mice (n=3 each); (D)Representative images of immunohistochemistry (IHC) staining showing IL-6 and IL-1β in the heart tissue from ethanol-exposed USP53^CKO^ mice and control mice; (E) IHC staining showing pMLKL and Cleaved caspase3 in the heart tissue from indicated mice. Results are expressed as the mean±SD; *P<0.05, **P<0.01, ***P<0.001.
